# Regulatory Significance of Plastic Manufacturing Air Pollution Discharged into Terrestrial Environments and Real-Time Sensing Challenges

**DOI:** 10.1021/acs.estlett.2c00710

**Published:** 2023-01-20

**Authors:** Yoorae Noh, Li Xia, Nadezhda N. Zyaykina, Brandon E. Boor, Jonathan H. Shannahan, Andrew J. Whelton

**Affiliations:** † Lyles School of Civil Engineering, Purdue University, 550 Stadium Mall Drive, West Lafayette, Indiana 47907, United States; ‡ School of Health Sciences, Purdue University, 550 Stadium Mall Drive, West Lafayette, Indiana 47907, United States; § Division of Environmental and Ecological Engineering, Purdue University, 500 Central Drive, West Lafayette, Indiana 47907, United States; ∥ Lyles School of Civil Engineering, Purdue University, 550 Stadium Mall Drive, West Lafayette, Indiana 47907, United States; ⊥ School of Health Sciences, Purdue University, 550 Stadium Mall Drive, West Lafayette, Indiana 47907, United States; # Lyles School of Civil Engineering and Division of Ecological and Environmental Engineering, Purdue University, 550 Stadium Mall Drive, West Lafayette, Indiana 47907-2051, United States

**Keywords:** Air chemical monitoring, Volatile organic compounds (VOCs), Plastic lining, Low-cost VOC sensors, Emission factors

## Abstract

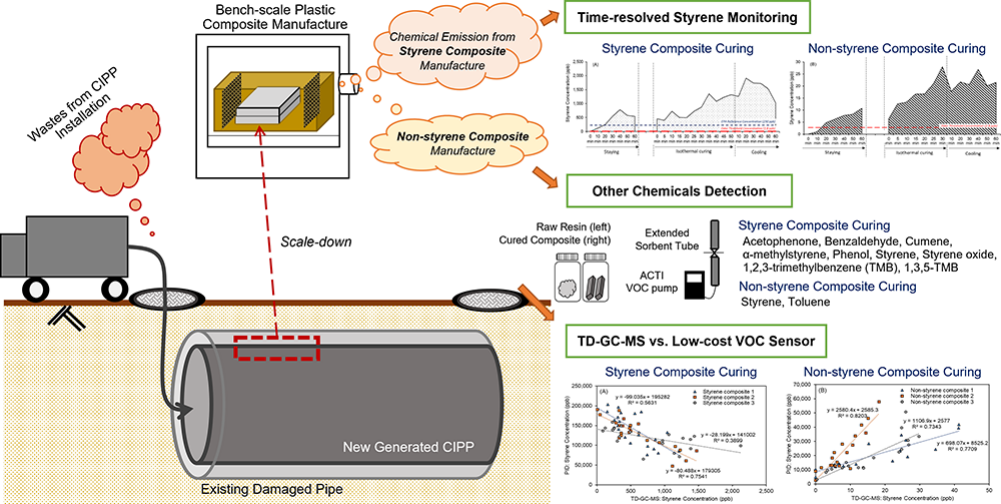

Cured-in-place-pipe (CIPP) is an onsite plastic manufacturing technology used in the U.S. and has not been evaluated for regulatory compliance with federal air pollution laws. The practice involves the discharge of manufacturing waste into the environment. The study goal was to estimate the magnitude of volatile organic compounds (VOCs) discharged into the atmosphere for styrene and nonstyrene composite manufacture and examine low-cost air monitoring sensor reliability. Time-resolved emission analysis revealed that VOC emission was not only isolated to the thermal curing period but also occurred before and after curing. In addition to the styrene monomer, other gas-phase hazardous air pollutants regulated under the *Clean Air Act* were also emitted. Based on typical CIPP installations, 0.9 to 16.6 U.S. tons of emitted VOCs were estimated for styrene CIPPs, and 0.09 to 1.6 U.S. tons of emitted VOCs were estimated for nonstyrene CIPPs. Because the number and size of CIPPs manufactured in a single community can vary, the total air pollution burden will significantly differ across communities. Low-cost VOC sensors commonly utilized near CIPP manufacturing activities did not accurately quantify styrene and should not be relied upon for that purpose. Up to several thousand-fold detection differences were observed. Regulatory evaluation of CIPP air pollution and PID sensor reliability assessments are recommended.

## Introduction

In response to buried infrastructure repair challenges, new volatile organic compound (VOC) and hazardous air pollutant (HAP) sources have emerged.^1^ The sources are companies that manufacture plastic (polymer) composite cured-in-place-pipes (CIPPs). CIPPs are being created in communities across Asia, Europe, Oceana, and North America. CIPP manufacturing is carried out by setting up a temporary worksite, inserting a styrene- or nonstyrene resin saturated tube into the damaged pipe, and polymerizing the resin into new plastic. VOCs and HAPs from the resin and manufacturing byproducts are emitted into the air without being captured or destroyed during the CIPP setup, curing, and cooling periods. Pollutant emission into ambient air is encouraged by industry (Table S1 and Figure S1).^2−4^

In the U.S., HAP emissions from polymer composite manufacturing operations like CIPP are regulated under federal law, but CIPP companies have not been evaluated by federal or state regulators.^5−7^ In particular, the *Clean Air Act* regulates the emission of specific HAPs into ambient air (Table S2). *National Emission Standards for Hazardous Air Pollutants (NESHAP)*(7) specifically apply to plastic composite manufacturing and classify sources based on their total HAP emitted per year. In 2022, the air pollution regulator for the U.S. District of Columbia began looking into VOC and HAP emissions but found information about emissions was lacking.^8−10^ The lack of HAP emission data from CIPP companies and projects has inhibited air pollution regulatory attention.^11,12^

Some CIPP manufacturing air sampling campaigns have been conducted but primarily focused on the HAP styrene. When styrene resins have been used, gas-phase concentrations in excess of 1,000 ppm were found before and during manufacture in ambient air.^3,13−18^ For the most popular thermal manufacturing practice, the atmosphere contained greater than 4,000 ppm styrene (S1).^15^ One investigation estimated that 6 to 33 U.S. tons of volatile chemicals (nonspecific) were discharged per past CIPP sewer projects (Table S3).^15^ If greater than 10 tons of HAP/year were emitted, a CIPP company would be classified as a major source according to the *NESHAP*. Numerous nonstyrene HAPs and other VOCs not listed on CIPP resin safety data sheets were found in the resins and emitted into air during manufacture.^3,4,15−22^ To compare HAP emission to *NESHAP* thresholds, time-resolved chemical emission data are needed.

Analytical challenges and lack of sensor reliability assessments have raised questions about CIPP manufacturing air pollution. The atmospheres created have prompted high styrene gas-phase concentrations that inhibited the detection of other VOCs present.^17,18,23^ The reliability of low-cost photoionization detector (PID) sensors for quantifying styrene concentration has not been evaluated.^13,15,17,19,24−33^ Despite this, one of the largest CIPP companies in the world claimed “... [the PID] can provide an accurate reading of styrene concentration on the job site as well as at manufacturing operations”.^34^ Some researchers have claimed that “PID measurements of total VOCs are ... an acceptable approximation of the styrene concentration”.^35^ Data was not found to support these statements for CIPP activities. Previous CIPP field studies revealed that a PID sensor over- and underestimated styrene gas-phase levels from 10- to 1,000-fold.^15,19^ U.S. occupational safety agencies have warned that PID sensors may provide erroneous results due to temperature, humidity, and calibration.^36−38^ U.S. agencies such as NIOSH and OSHA have recommended styrene quantification using sorbent tubes and/or canisters followed by gas chromatography–mass spectrometry (GC-MS) analysis.^17,39,40^ Evidence is needed to understand the utility of PID sensors for CIPP air pollution assessments.

The study goal was to better understand time-resolved VOC emissions during the thermal manufacture of CIPP and evaluate portable real-time PID sensor reliability. Specific objectives of this study were to (1) determine the magnitude of styrene emitted during different phases of composite manufacture, (2) investigate whether chemicals other than styrene were present in gas-phase emissions, (3) evaluate the accuracy of a portable real-time PID sensor used at CIPP manufacturing sites, and (4) estimate chemical emissions during full-scale CIPP projects.

## Materials and Methods

### Plastic Composite Manufacture

Unsaturated polyester resin and vinyl hybrid resin were used as a styrene and nonstyrene resin, respectively. Composite plates (10.16 cm × 10.16 cm × 0.6 to 0.8 cm) were manufactured following resin manufacturer recommendations (S1).^41^ Two-, four-, and six-layered thick uncured resin-impregnated fabrics (up to 2.5 cm) were prepared and polymerized using a dry-heating curing method. The thicknesses of the lab-manufactured composites in the present study were similar to that of field manufactured CIPPs (0.6 to 8.6 cm) (Figure S2).^41−43^ Composites were thermally cured in an electropolished stainless steel environmental test chamber (ETC),^18^ which was located inside an oven (Figure S3). The resin manufacturer’s recommended composite curing be conducted at 65.6 °C for 50 min (styrene composite) and at 82.2 °C for 30 min (nonstyrene composite) mimicking the actual CIPP installation. Temperature profiles and relative humidity in the ETC were measured (S2). Three composites were manufactured, and three replicate experiments were performed per curing conditions.

### Emission Monitoring and Analysis

#### VOC Emission Monitoring Strategy before, during, and after Styrene- and Nonstyrene Composite Manufacture

Air testing and composite monitoring experiments were conducted in the sampling chamber-ETC setup (Figure S3A and Figure S4A). Composites were cured in the ETC, and air samples were collected from the sampling chamber. The larger sampling chamber provided an experimental basis for further toxicological evaluation of the volatile chemical emissions using animal models. All chambers and connecting stainless steel tubing were tightly sealed. Sampling was conducted every 5 to 10 min. Samples were collected for 30 s across four composite manufacture stages: (1) leaving the composite inside the oven for 1 h at ambient temperature (21 to 23 °C, “Staying”), (2) heating up to set-point temperatures (“Preheating”), (3) maintaining a constant oven temperature for 50 min with 65.6 °C for styrene composites and 30 min with 82.2 °C for nonstyrene composites (“Isothermal curing”), and (4) composite cooling after turning off the oven for 1 h (“Cooling”). The ETC was flushed with ultrahigh purity (UHP) air (1.4 L/min flow rate). The airflow was selected based on the CIPP area specific airflow rate at field installation conditions (S3). In this case, two-layered thin styrene- and nonstyrene composites were used.

Because styrene was only detected in the sampling chamber, VOC sampling was also performed directly from the ETC exhaust to aid in capturing nonstyrene compounds present (Figure S3B and Figure S4B). Four-layered thick composites were used to increase the source materials and detect the nonstyrene compounds. VOC sampling (10 or 15 min) was conducted in four exposure stages, selected based on sampling chamber monitoring results: (1) middle of isothermal curing (i.e., 10 min after starting isothermal hold), (2) end of isothermal curing (15 min before halting heating), (3) during cooling phase 1 (10 min after halting heating), and (4) during cooling phase 2 (35 min after halting heating). VOC concentrations were monitored to verify background residual contamination before starting each composite emission monitoring. Control tests and equipment decontamination activities were conducted (S4).

#### Sampling and Analytical Approach

Approximately 25 mL (30 s sampling), 0.5 L (10 min), and 0.75 L (15 min) of air samples were collected using an ACTI VOC vacuum pump (Markes International, Inc., CA) with a 50 mL/min flow rate and sorbent tubes packed with quartz wool, TenaxTA, and Carbograph 5TD. For multiple chemical air monitoring experiments, an extended adsorption tube (i.e., two adsorption tubes connected in series) was used to increase the adsorption surface area (Figure S4B). The prepared sorbent tube samples were analyzed using a Unity 2 Series thermal desorption (TD) system (Markes International, Inc., CA) in conjunction with a GC (2010-Plus, Shimadzu, Inc., MD) and an MS (TQ8040, Shimadzu, Inc., MD). Analyzed concentrations (mixing ratios) are presented in units of “ppb” in the air or emitted compound mass per composite surface area ‘mg/cm^2^_composite_’. The detailed analytical methods, quantification, and tube decontamination are described in S5.

A calibrated PID sensor measured the total VOC signal (ppbRae 3000 PID, *f*_*s*_ = 1/60 s^–1^, RAE Systems, 10.6 eV lamp, CA) of the ETC exhaust. The PID sensor was calibrated before each experiment using UHP air and 10 ppm isobutylene. The PID sensor response was converted to styrene using a response factor of 0.43 provided by the manufacturer. The data was analyzed for statistical significance by applying linear regression analysis with a Type I error of 0.05 using statistics package IBM SPSS Statistics 21 (S5).

Styrene emission factors (EF, mg/kg_resin_) were calculated for each manufacturing stage using a mass conservation formula, treating the ETC and sampling chamber as completely mixed flow reactors (Figures S5 and S6).

## Results and Discussion

### Time-Resolved Styrene Emission during Composite Manufacture

**Figure 1:**
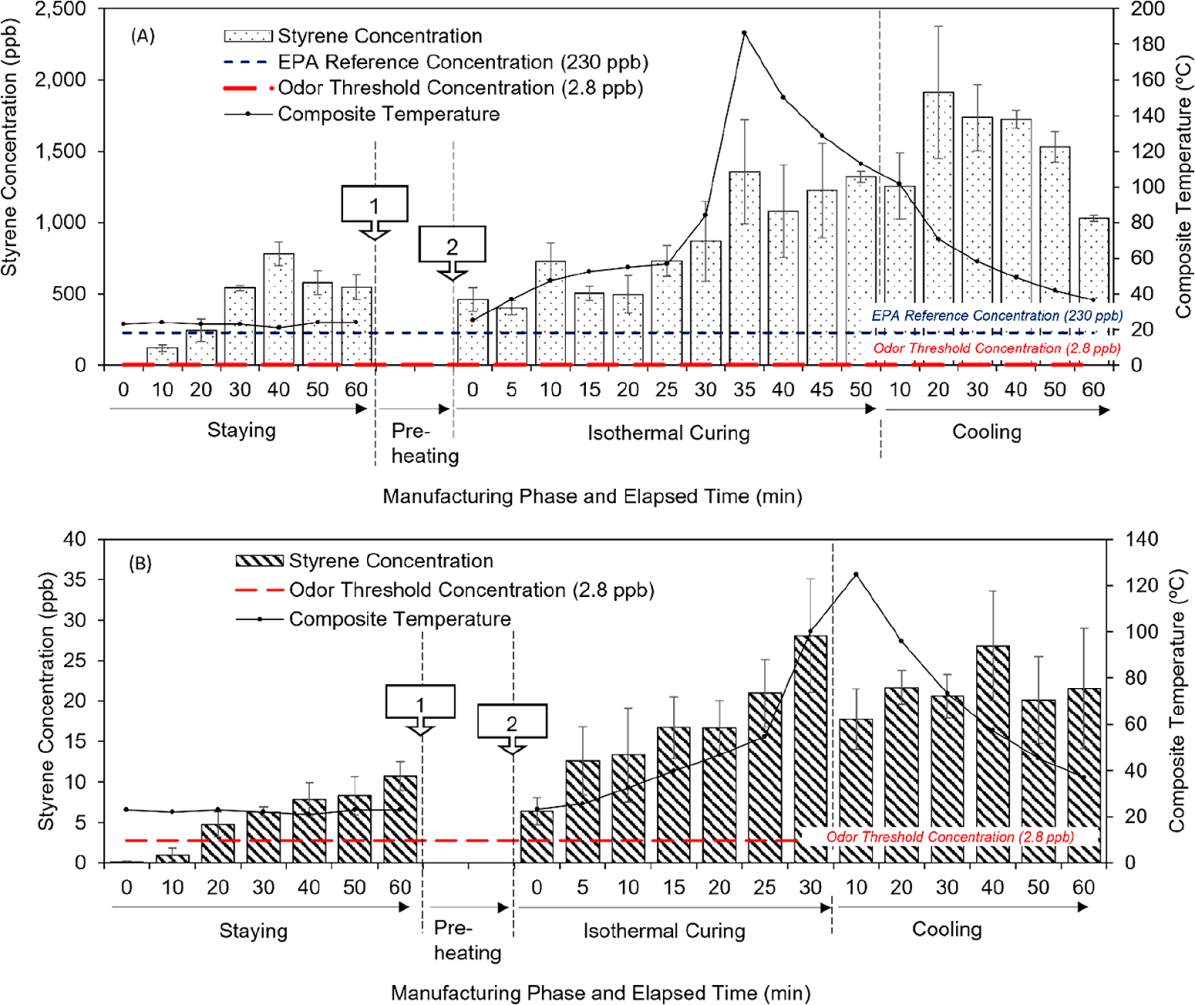
Styrene gas-phase concentrations measured using TD-GC-MS throughout curing for (A) styrene composites (two-layered) and (B) nonstyrene composites (two-layered) (*n* = 3/assessment) with respect to composite temperature (°C). At point [1], the uncured resin fabric was removed from the heating chamber, and heating was started to a certain set-point temperature. After the heating chamber reached the heating temperature (65.6 °C for styrene curing and 82.2 °C for nonstyrene curing), the uncured resin fabric was put into the chamber at point [2].

Styrene emission was the initial focus of the author’s initial evaluation because not only was styrene a primary emission component produced from composite manufacturing but also the authors and a U.S. government agency previously hypothesized styrene’s magnitude inhibited the detection of other low concentration substances.^17,18^ Composites created in the present study were scaled down versions of field CIPPs. Both styrene and nonstyrene composites emitted styrene during manufacture. The emission magnitude was much greater for the styrene-resin composite than the nonstyrene composite (Figure 1). Styrene levels reached 1,913 ± 920 ppb (normalized styrene concentration: 4 ± 1 × 10^–3^ mg/cm^2^_composite_) for two-layer styrene composite curing and 28 ± 14 ppb (6 ± 3 × 10^–5^ mg/cm^2^_composite_) for two-layer nonstyrene composite curing. Prior chemical analysis revealed that the nonstyrene resin manufacturer contaminated their product with a low amount of styrene (<1 wt %).^41^ The greatest styrene concentration was detected during styrene composite cooling, not during the staying or isothermal curing phases. The lowest styrene concentration for the nonstyrene composite was found during the staying phase.

For both manufacturing operations, the composite temperature exceeded the manufacturer recommended temperature both in the isothermal curing and cooling phase. The temperature exceedance issue was also observed during field CIPP installations.^19^ This can be attributed to exothermic reactions during polymerization.^40^ As the composite temperature increased, the emitted styrene concentration also increased (both *p*-values < 0.05) (Figure S6). Since styrene becomes more volatile at higher temperature, its emission could also increase after the isothermal curing phase. A U.S. government agency previously found that, at CIPP field sites, styrene levels were greatest during the cooling phase, not during the isothermal curing phase.^17^ While the cooling phase was only 1 h in the present study, others found styrene was still volatilizing from CIPPs after 12 h when the study was halted.^18,25,28^

### Other HAPs and VOCs Not Disclosed on Safety Data Sheets Were Emitted into the Air

To better understand the emission of nonstyrene compounds from CIPP manufacturing operations, double the amount of resin was cured into composites. During styrene composite manufacture, nine VOCs (five HAPs) were emitted into the air (Table S4, Table S5, and Figure S7). Except for styrene, none of the compounds were listed in the resin safety data sheet.^44^ VOCs found were styrene oxidation products (acetophenone, benzaldehyde, styrene oxide), resin solvent/intermediates (cumene, α-methylstyrene, 1,2,3-TMB, 1,3,5-TMB), and phenol. These include carcinogenic compounds, endocrine disruptors, and chemical irritants.^45−48^

For the nonstyrene composite, except for a comparatively low amount of styrene, no VOCs were detected in the air. However, a variety of VOCs and HAPs were identified in the uncured resin [methacrylic acid (9.96 × 10^3^ mg/kg) > styrene (6.90 × 10^3^ mg/kg) > toluene (2.77 × 10^3^ mg/kg) and five other compounds].^41^ A follow-up experiment where nonstyrene resin and nonstyrene composite samples were stored in closed vials for 24 h at room temperature revealed that the HAP toluene was emitted into air (S7). The author’s inability to capture and quantify VOCs was likely associated with the sampling and analysis methods, as well as physical properties (i.e., high volatility and low retention time) (Table S6).

### Estimated Field-Scale CIPP VOC Emissions Were Substantial

To estimate VOC emissions in full-scale CIPP installations, styrene and total VOC emission magnitudes were calculated. For both styrene and nonstyrene composite manufacture, the amount of styrene increase changed abruptly with increased composite temperature (Figure S8 and Figure 1). It is known that polymerization rates increase when temperatures increase rapidly, but the adsorption binding energy at the surface of each composite can be thermodynamically weakened,^49^ and VOC “emission” occurs at the surface.

The total styrene EF for the laboratory-based styrene composite (1 h staying + 50 min isothermal curing + 1 h cooling) was 3,777 ± 132 mg per kg resin_total_ (Table S7). For reference, about 24,600 to 454,000 kg of resin has been used for some sewer pipe diameters of 0.6 to 2.4 m (Table S3).^18,41,50^ For the present study, the VOC mass emitted into the air was 3.31 wt % of the initial resin_total_ (i.e., resin mixture). Therefore, approximately 815 to 15,030 kg of VOC and 93 to 1,715 kg of styrene were estimated to be emitted per one-time CIPP projects in the field. For the nonstyrene composite, approximately 80 to 1,450 kg of VOC and 1.4 to 26 kg of styrene per nonstyrene CIPP project were estimated. Follow-up experiments revealed a proportional relationship between styrene emission and the mass of resin used (Figure S9).

### PID Sensors Did Not Predict Styrene Air Concentrations for Composite Manufacture

The PID sensor overestimated styrene levels by a factor of 40 to 3,500 in the present study, and increasing styrene concentrations caused PID sensor signals to both increase and decrease. The atmosphere created by the styrene resin composite manufacturing process prompted the PID sensor signal to increase, but the signal decreased for the nonstyrene composite curing atmosphere (both *p*-values < 0.05) (Figure S10, Figure S11, and Table S8). Because humidity was low and temperature was similar, these differences may be due to the PID sensor responding to other VOCs and the predominance of styrene (S8). Field use of another one of these devices underestimated and overestimated styrene levels by a factor of 10s to 1,000s at CIPP air pollution discharge locations.^19^

### Implications

Study results can now help scientists and regulators begin to estimate VOC and HAP emission magnitudes from CIPP manufacturing projects. The author’s analytical approach (i.e., multiple sorbent tubes in series) enabled styrene and nonstyrene VOCs and HAPs to be captured. While styrene was the HAP emitted in greatest magnitude, several other compounds released from both resins were HAPs. Therefore, to estimate the total amount of VOC and HAP emitted by CIPP manufacture, the totality of chemical emissions, not just styrene, must be considered. These nonstyrene components of the emission mixture have shown to be responsible for toxicological effects (S9).^51^ PID sensors should not be used to estimate air pollution magnitude, gas-phase styrene concentrations, and the capabilities of these sensing technologies, and their limitations require scrutiny (S9).^52^

Following the current study, a variety of additional studies are recommended (S9). Composite manufacture in the present study was conducted without steam or hot water, but the presence of water may prompt different VOC emission profiles and magnitudes. The role of the resin mass, pipe size, extent of damage, types of inner and outer CIPP coatings, and other factors in the field should be evaluated for quantifying VOC emission. In the present work, the emission factor was calculated based on comparatively brief manufacturer recommended heating duration (1 h). However, in the field, heating of large diameter CIPPs can occur for more than 22 h, which does not include the subsequent 24 h of cooling.^50^ These durations may prompt significant amounts of VOC and HAP emission. In some cases, there have been more than 15 CIPPs manufactured in a single city over a few months’ period, so the local and regional air pollution impacts should be estimated.

In the U.S., the *Clean Air Act* was created to protect public health and public welfare and to regulate HAP emissions. Typically, polymer composite manufacturing is conducted at a fixed location (i.e., boat, aircraft fuselage) and is regulated by a state’s primacy agency. Like those sources, CIPP companies handle raw materials at a fixed facility and must comply with regulatory HAP emission standards. However, CIPP companies emit HAPs outside their facility at multiple locations (i.e., single municipality or neighborhoods) and also travel to different states emitting HAPs into different regulatory jurisdictions. Federal and state regulators do not track how much air pollution CIPP companies emit in each state. Results of the present study can begin to enable regulators to make those estimates.

As the number of CIPP manufacturing projects increases, the magnitude of annual HAP emissions from CIPP companies will increase. A review of CIPP manufacturers similar to the U.S. EPA’s 1990s review of boat manufacturers^53^ has not yet been conducted. For reference, the U.S. EPA (1996) reported that 144 fiberglass boat manufacturers emitted about 6,300,000 kg of styrene/year, which was 94.7% of their measured total HAP emissions.^54^ Like boat manufacturers, regulatory agencies should evaluate air pollution monitoring, permitting, and control operations for CIPP manufacturers.^55^ Other helpful information should be considered: CIPP resin usage per project, resin chemical composition (includes materials not listed on SDSs), emission magnitudes, and emission composition (includes manufacture byproducts). Emission monitoring, controls, and greater environmental regulatory oversight could help protect air quality where buried infrastructure is needing repair.

## Supporting Information Available

Additional CIPP installation and incident information, experimental materials and methods for air monitoring and headspace analysis, and estimation of VOC levels compared to styrene levels (PDF)

## Notes

^○^ Email: noh18@purdue.edu.

## Notes

^◆^ Email: xia104@purdue.edu.

## Notes

^▲^ Email: nzyaykina@purdue.edu.

## Notes

^■^ Email: bboor@purdue.edu.

## Notes

^●^ Email: jshannah@purdue.edu.

## References

[ref1] Stratview Research Cured-in-Place Pipe Market Size, Share, Trend, Forecast, Competitive Analysis, and Growth Opportunity: 2022–2027; 2022. Available at https://www.stratviewresearch.com/287/Cured-in-Place-Pipe-CIPP-Market.html (accessed 2023-01).

[ref2] Kampbell N. E. Guideline for the Use and Handling of Styrenated Resins in Cured-In-Place Pipe; National Association of Sewer Service Companies (NASSCO) CIPP Council: Hilliard, OH, 2009.

[ref3] Matthews E.; Matthews J.; Alam S.; Eklund S. NASSCO CIPP Emissions Phase 2: Evaluation of Air Emissions from Polyester Resin CIPP with Steam Cure: Final Report; For National Association of Sewer Service Companies, Inc. (NASSCO, Inc.), Louisiana Tech University: Ruston, LA, 2020.

[ref4] National Association of Sewer Service Companies, Inc. (NASSCO, Inc.) Guideline for the Safe Use and Handling of Styrene-Based Resins in Cured-in-Place Pipe; Marriottsville, MD, 2020.

[ref5] U.S. Environmental Protection Agency (USEPA) Clean Air Act Standards and Guidelines for Foam, Fiber, Plastic and Rubber Products; Washington, DC, 2022. Available at https://www.epa.gov/stationary-sources-air-pollution/clean-air-act-standards-and-guidelines-foam-fiber-plastic-and (accessed 2023-01).

[ref6] U.S. Environmental Protection Agency (USEPA) Polymer Manufacturing Industry: Standards of Performance for Volatile Organic Compound (VOC) Emissions; Washington, DC, 2022. Available at https://www.epa.gov/stationary-sources-air-pollution/polymer-manufacturing-industry-standards-performance-volatile (accessed 2023-01).

[ref7] U.S. Environmental Protection Agency (USEPA) 40 CFR Part 63 National Emission Standards for Hazardous Air Pollutants: Reinforced Plastic Composites Production; Proposed Rule; Washington, DC, 2001.

[ref8] Berlin M. DC Water plans summer start for Soapstone sewer work as the community voices concerns; Forest Hills Connection: Washington, DC, January 10, 2022. Available at https://www.foresthillsconnection.com/news/after-the-community-raised-air-pollution-concerns-dc-water-is-delaying-soapstone-sewer-work/ (accessed 2023-01).

[ref9] Share M. L. Opinion: Upcoming Soapstone sewer work will use a method that’s toxic to humans and the environment. There is another way; Forest Hills Connection: Washington, DC, December 6, 2021. Available at https://www.foresthillsconnection.com/news/opinion-upcoming-soapstone-sewer-work-will-use-a-method-thats-toxic-to-humans-and-the-environment-there-is-another-way/ (accessed 2023-01).

[ref10] Department of Energy & Environment (DOEE) Apply for an Air Pollutant Permit; Washington, DC, 2022. Available at https://doee.dc.gov/node/9212 (accessed 2023-01).

[ref11] Noh Y.; Shannahan J. H.; Hoover A. G.; Pennell K. G.; Weir M. H.; Whelton A. J. W. Bystander Chemical Exposures and Injuries Associated with Nearby Plastic Sewer Pipe Manufacture: Public Health Practice and Lessons. J. Environ. Health 2022, 85, 22. PMC10512871 37736399

[ref12] Morales A. C.; Tomlin J. M.; West C. P.; Rivera-Adorno F. A.; Peterson B. N.; Sharpe S. A.; Noh Y.; Sendesi S. M.; Boor B. E.; Howarter J. A.; Moffet R. C.; et al. Atmospheric emission of nanoplastics from sewer pipes repair. Nat. Nanotechnol. 2022, 17, 1171 10.1038/s41565-022-01219-9. 36203091

[ref13] AirZone, Inc. A Report on the Monitoring of Styrene in Toronto Homes During the Cured in Place Pipe (CIPP) Process for Sewer Pipe Rehabilitation by Insituform. Project 041-6742; Toronto, CAN, 2001.

[ref14] Agency for Toxic Substances and Disease Registry (ATSDR) Health Consultation. Division of Health Assessment and Consultation; Health Consultation: Schlitz Park Office Building, Milwaukee, WI, 2005.

[ref15] Teimouri Sendesi S. M.; Ra K.; Conkling E. N.; Boor B. E.; Nuruddin M.; Howarter J. A.; Youngblood J. P.; Kobos L. M.; Shannahan J. H.; Jafvert C. T.; et al. Worksite chemical air emissions and worker exposure during sanitary sewer and stormwater pipe rehabilitation using cured-in-place-pipe (CIPP). Environ. Sci. Technol. Lett. 2017, 4 (8), 325–333. 10.1021/acs.estlett.7b00237.

[ref16] Ajdari E. B. Volatile organic compound (VOC) emission during cured-in-place-pipe (CIPP) sewer pipe rehabilitation; University of New Orleans: New Orleans, LA, 2016.

[ref17] National Institute for Occupational Safety and Health (NIOSH) Evaluation of Exposures to Styrene During Ultraviolet Cured-in-Place-Pipe Installation; HHE Report No. 2018-0009-3334; NIOSH Health Hazard Evaluation Report; U.S. Department of Health and Human Services, Centers for Disease Control and Prevention: Morgantown, WV, 2019; 10.26616/nioshhhe201800093334revised032019.

[ref18] Teimouri Sendesi S. M.; Noh Y.; Nuruddin M.; Boor B. E.; Howarter J. A.; Youngblood J. P.; Jafvert C. T.; Whelton A. An Emerging Mobile Air Pollution Source: Outdoor Plastic Liner Manufacturing Sites Discharge VOCs into Urban and Rural Areas. Environ. Sci. Process Impacts 2020, 22, 1828 10.1039/D0EM00190B. 32852018

[ref19] Ra K.; Sendesi S. M. T.; Nuruddin M.; Zyaykina N. N.; Conkling E. N.; Boor B. E.; Jafvert C. T.; Howarter J. A.; Youngblood J. P.; Whelton A. J. Considerations for emission monitoring and liner analysis of thermally manufactured sewer cured-in-place-pipes (CIPP). J. Hazard. Mater. 2019, 371, 540–549. 10.1016/j.jhazmat.2019.02.097. 30877867

[ref20] Whelton A. J.; Ra K.; Teimouri Sendesi S. M.; Nuruddin M.; Li X.; Howarter J. A.; Youngblood J. P.; Jafvert C. T.; Zyaykina N. N. Contaminant Release from Storm Water Culvert Rehabilitation Technologies: Understanding Implications to the Environment and Long-Term Material Integrity. (U.S. Federal Highway Administration Transportation Pooled Fund (TPF)-5(339)); Purdue University: West Lafayette, IN, 2019. Available at 10.5703/1288284317089.

[ref21] Nuruddin M. P.; Mendis G.; Ra K.; Sendesi S. M. T.; Futch T.; Goodsell J.; Whelton A. J.; Youngblood J. P.; Howarter J. A. Evaluation of the physical, chemical, mechanical, and thermal properties of steam-cured PET/polyester cured-in-place pipe. J. Compos. Mater. 2019, 53 (19), 2687–2699. 10.1177/0021998319839132.

[ref22] National Institute for Occupational Safety and Health (NIOSH) LeBouf R. F.; Burns D. A.; Ranpara A.; Kobos L. Evaluation of Exposures to Styrene during Cured-in-place Pipe Liner Preparation and during Pipe Repairs using Hot Water and Steam; Report 2019-0080-3379; U.S. Department of Health and Human Services, Centers for Disease Control and Prevention, NIOSH, Health Hazard Evaluation: Morgantown, WV, 2021. Available at https://www.cdc.gov/niosh/hhe/reports/pdfs/2019-0080-3379.pdf (accessed 2023-01).

[ref23] Knight M. A.; Ioannidis M. A.; Salim F.; Górecki T.; Pivin D. Health Risks Assessment from Cured-in-Place Pipe Lining Fugitive Styrene Emissions in Laterals. Journal of Pipeline Systems Engineering and Practice 2023, 14, 04022056 10.1061/(ASCE)PS.1949-1204.0000690.

[ref24] Najafi M.; Sattler M.; Schug K.; Kaushal V.; Iyer G. Evaluation of Potential Release of Organic Chemicals in the Steam Exhaust and Other Release Points during Pipe Rehabilitation Using the Trenchless Cured-In-Place Pipe (CIPP) Method, Final Report; Prepared for NASSCO, Inc., Marriottsville, MD, 2018.

[ref25] RIVM (Rijksinstituut voor Volksgezondheid en Milie, Netherlands National Institute for Public Health and the Environment) Sewer renovation with stocking methods: backgrounds in the information sheet; Report Number 609021038/2006; Amsterdam, NED, 2006.

[ref26] Bauer G., Styrene: An overview—An awareness; Underground Infrastructure Research International Conference and Trenchless Technology Road Show: Waterloo, CAN, 2012.

[ref27] Dusseldorp A.; Schols E. Rioolrenovatie met kousmethoden-Achtergronden bij het informatieblad; RIVM rapport 609021038/2006; RIVM-Rijksinstituut voor Volksgezondheid en Milieu: Bilthoven, Amsterdam, NED, 2006.

[ref28] Quantor Development of styrene vapour during the renovation of sewer pipes; Report AK-06-006; RIVM (Rijksinstituut voor Volksgezondheid en Milie; Netherlands National Institute for Public Health and the Environment): Amsterdam, NED, 2006.

[ref29] Circle Safety and Health Consultants, LLC. (CSHC) Industrial Hygiene Evaluation: CIPP – Styrene Exposure; Prince William County Service Authority: Woodbridge, VA, 2017.

[ref30] EHS-Alaska, Inc. Styrene Exposure During Hot Water Cured-in-Place Pipe Lining; Eagle River, AK; Anchorage Water and Wastewater Utility: Anchorage, AK, 2018.

[ref31] Ganley S. Subject: RE: Question about your Styrene Air Testing Data, Email from PipeWorks (Simon Ganley) to Purdue University (Andrew Whelton); November 18, 2018.

[ref32] Londer D. How to successfully install cured in place pipe (CIPP) lining. Proceedings for the Institute for Public Works Engineering Australiasa (IPWEA) Conference; Rotorua, NZL, June 20–22, 2018.

[ref33] PBSUSA Air Quality Summary Report Styrene Exposure Monitoring, Portland, Oregon, PBS Project 25024.006, Phase 0001; Portland, OR, 2018.

[ref34] National Association of Sewer Service Companies, Inc. (NASSCO, Inc.) How to Measure Styrene on a CIPP Jobsite; Marriottsville, MD, 2021.

[ref35] Howell J. M.; Matthews E.; Matthews J.; Alam S.; Bednar A.; Laber C.; Eklund S. Styrene Emissions in Steam-Cured CIPP: A Review and Comparison of Multiple Studies. J. Pipeline Syst. Eng. Pract. 2022, 13 (1), 04021071 10.1061/(ASCE)PS.1949-1204.0000620.

[ref36] LeBouf R. F.; Coffey C. C. Effect of interferents on the performance of direct-reading organic vapor monitors. J. Air Waste Manag. Assoc. 2015, 65 (3), 261–269. 10.1080/10962247.2014.986308. 25947122 PMC4657743

[ref37] LeBouf R. F.; Slaven J. E.; Coffey C. C. Effect of calibration environment on the performance of direct-reading organic vapor monitors. J. Air Waste Manag. Assoc. 2013, 63 (5), 528–533. 10.1080/10962247.2013.772926. 23786144

[ref38] Coffey C.; LeBouf R.; Lee L.; Slaven J.; Martin S. Effect of calibration and environmental condition on the performance of direct-reading organic vapor monitors. J. Occup. Environ. Hyg. 2012, 9 (11), 670–680. 10.1080/15459624.2012.725015. 23016630

[ref39] National Institute for Occupational Safety and Health (NIOSH). Volatile organic compounds, C1 to C10, Canister method: Method 3900. In NIOSH manual of analytical methods (NMAM), 5th ed.; Ashley K., O’Connor P. F., Eds.; Washington, DC, 2018. Available at https://www.cdc.gov/niosh/nmam/pdf/3900.pdf (accessed 2023-01).

[ref40] Occupational Safety and Health Administration (OSHA). OSHA Sampling and Analytical Methods; US Department of Labor, Occupational Safety and Health Administration: Washington, DC, 1991. Available at http://www.osha.gov/dts/sltc/methods/index.html (accessed 2023-01).

[ref41] Noh Y.; Odimayomi T.; Sendesi S. M. T.; Younblood J. P.; Whelton A. J. Environmental and Human Health Risks of Plastic Composites can be Reduced by Optimizing Manufacturing Conditions. J. Clean. Prod. 2022, 356, 131803 10.1016/j.jclepro.2022.131803.

[ref42] North Carolina Department of Transportation (NCDOT). NCDOT Pipe Liner Manual; Raleigh, NC, 2019. Available at https://connect.ncdot.gov/resources/hydro/Hydraulics%20Memos%20Guidelines/NCDOT%20Pipe%20Liner%20Manual.pdf (accessed 2023-01).

[ref43] Allouche E.; Alam S.; Simicevic J.; Sterling R.; Condit W.; Headington B.; Downey D. A retrospective evaluation of cured-in-place pipe (CIPP) used in municipal gravity sewers; U.S. Environmental Protection Agency: Washington, DC, 2012.

[ref44] Interplastic Corporation. Safety Data Sheet: CIPP ISO Resin; St. Paul, MN, 2015. Available at https://pipeliningsupply.com/wp-content/uploads/2014/09/MSDS_Poly.pdf (accessed 2023-01).

[ref45] National Toxicology Program. NTP 15th report on carcinogens; Department of Health and Human Services (HHS): Washington, DC, 2021. Available at https://ntp.niehs.nih.gov/whatwestudy/assessments/cancer/roc/index.html (accessed 2023-01). 42475452

[ref46] National Institute for Occupational Safety and Health (NIOSH). NIOSH Pocket Guide to Chemical Hazards; Washington, DC, 2022. Available at https://www.cdc.gov/niosh/npg/npgsyn-a.html (accessed 2023-01).

[ref47] U.S. Environmental Protection Agency (USEPA). Final Second List of Chemicals for Tier 1 under the Endocrine Disruptor Screening Program; Washington, DC, 2014. Available at https://www.epa.gov/endocrine-disruption/final-second-list-chemicals-tier-1-under-endocrine-disruptor-screening-program (accessed 2023-01).

[ref48] U.S. Environmental Protection Agency (USEPA). Initial List of Hazardous Air Pollutants with Modifications; Washington, DC, 2022. Available at https://www.epa.gov/haps/initial-list-hazardous-air-pollutants-modifications (accessed 2023-01).

[ref49] Fedorova A. A.; Ulitin M. V. Adsorption of styrene at a binary solution-gas interface. Russ. J. Phys. Chem. 2011, 85 (10), 1810–1813. 10.1134/S0036024411100050.

[ref50] Matthews J. C. Large-diameter sewer rehabilitation using a fiber-reinforced cured-in-place pipe. Pract. Period. Struct. Des. Cons. 2015, 20 (2), 04014031 10.1061/(ASCE)SC.1943-5576.0000231.

[ref51] Kobos L.; Teimouri Sendesi S. M.; Whelton A. J.; Boor B. E.; Howarter J. A.; Shannahan J. In vitro toxicity assessment of emitted materials collected during the manufacture of water pipe plastic linings. Inhal. Toxicol. 2019, 31 (4), 131–146. 10.1080/08958378.2019.1621966. 31187656 PMC6639800

[ref52] Williams R., Kaufman A., Garvey S. Next Generation Air Monitoring (NGAM) VOC Sensor Evaluation Report. U.S. Environmental Protection Agency; Washington, DC, 2015. Available at https://cfpub.epa.gov/si/si_public_record_report.cfm?Lab=NERL=308114 (accessed 2023-01).

[ref53] U.S. Environmental Protection Agency (USEPA) 85 FR 15960 - National Emission Standards for Hazardous Air Pollutants: Boat Manufacturing and Reinforced Plastic Composites Production Risk and Technology Review; Office of the Federal Register, National Archives and Records Administration: Washington, DC, EPA/600/R-15/122 (NTIS PB2015-105133), 2015. Available at https://www.govinfo.gov/app/details/FR-2020-03-20/2020-04661 (accessed 2023-01).

[ref54] Kong E. J.; Bahner M. A.; Turner S. L. Assessment of styrene emission controls for FRP/C and boat building industries; United States Environmental Protection Agency, Research and Development: Washington, DC, 1996. Available at https://www3.epa.gov/airtoxics/coat/rein/finalrpt.pdf (accessed 2023-01).

[ref55] U.S. Environmental Protection Agency (USEPA) Final Risk and Technology Review: Boat Manufacturing and Reinforced Plastics Manufacturing National Emissions Standards for Hazardous Air Pollutants; Washington, DC, 2020. Available at https://www.epa.gov/stationary-sources-air-pollution/final-risk-and-technology-review-boat-manufacturing-and-reinforced (accessed 2023-01).

