# Supplementary material for: Regulatory Significance of Plastic Manufacturing Air Pollution Discharged into Terrestrial Environments and Real-Time Sensing Challenges
Source: Environ Sci Technol Lett. 2023 Jan 20;10(2):152–8. doi: 10.1021/acs.estlett.2c00710 (PMC9933524; doi:10.1021/acs.estlett.2c00710)
Supplement: Supplementary file 1 — ez2c00710_si_001.pdf [file ez2c00710_si_001.pdf]

Electronic Supporting Information

Regulatory Significance of Plastic Manufacturing Air Pollution Discharged into Terrestrial Environments and Real-Time Sensing Challenges

Yoorae Noh[1], Li Xia[2], Nadezhda N. Zyaykina[3], Brandon E. Boor[4], Jonathan H. Shannahan[5], and Andrew J. Whelton[6]*

[1] Lyles School of Civil Engineering, Purdue University, 550 Stadium Mall Drive, West Lafayette, Indiana USA 47907; noh18@purdue.edu

[2] School of Health Sciences, Purdue University, 550 Stadium Mall Drive, West Lafayette, Indiana USA 47907; xia104@purdue.edu

[3] Division of Environmental and Ecological Engineering, Purdue University, 500 Central Drive, West Lafayette, Indiana USA 47907; nzyaykina@purdue.edu

[4] Lyles School of Civil Engineering, Purdue University, 550 Stadium Mall Drive, West Lafayette, Indiana USA 47907; bboor@purdue.edu

[5] School of Health Sciences, Purdue University, 550 Stadium Mall Drive, West Lafayette, Indiana USA 47907; jshannah@purdue.edu

[6] Lyles School of Civil Engineering and Division of Ecological and Environmental Engineering, Purdue University, 550 Stadium Mall Drive, West Lafayette, Indiana USA 47907; T: (765) 494-2166; awhelton@purdue.edu

[*]Corresponding author: Professor, Lyles School of Civil Engineering and Division of Ecological and Environmental Engineering, Purdue University, 550 Stadium Mall Drive, West Lafayette, IN USA 47907-2051; T: (765) 494-2166; F: (765) 494-0395; E: ajwhelton@gmail.com; awhelton@purdue.edu

Contents

Table S1. List of CIPP associated air contamination incidents

Table S2. HAPs which are regulated by the Clean Air Act have previously been found in resins or discharged into air

Table S3. Example CIPP installation use in USA and other countries

Table S4. Multiple VOCs were emitted into air during and after styrene composite manufacture

Table S5. Public health and occupational exposure limits of gas phased chemicals

Table S6. Chemical volatility and retention time

Table S7. Emitted styrene mass loading during styrene and non-styrene composite manufacture

Table S8. Statistical summary of the gas-phase styrene concentration comparison measured by PID and TD-GC-MS during styrene and non-styrene composite manufacture

Figure S1. CIPP generated chemical exposure incidents across the world and countries where CIPP air emission studies were conducted.

Figure S2. Comparison of lab manufactured composites (bottom; white) and field generated CIPPs (top; blue): [A] thin layer and [B] thick layer

Figure S3. Experimental setup for emitted air collection from [A] sampling chamber and [B] stainless steel environmental test chamber

Figure S4. Experimental setup of composite curing and air monitoring for: (A) styrene monitoring in the sampling chamber and (B) multiple chemical monitoring in the ETC. The emission flow is presented as an arrow

Figure S5. Schematic for the material balance model for gas-phase styrene

Figure S6. Styrene gas-phase concentrations as measured with TD-GC-MS for (A) styrene composites (two-layered) and (B) non-styrene composites (two-layered) (n=3/assessment) and calculated styrene vapor pressure with respect to composite temperature (°C)

Figure S7. TD-GC-MS measured gas phased chemical concentrations and its volatility (i.e., vapor pressure, kPa at 25 °C) during and after cuing for styrene composite (4-layered)

Figure S8. Cumulative mass of gas-phase styrene emission normalized by manufacturing phase duration in the ETC for (A) styrene composites (two-layered) and (B) non-styrene composites (two-layered) manufacture

Figure S9. Gas phased styrene monitoring before, during, and after cuing for thick styrene composite (six-layered)

Figure S10. PIDs were not effective in predicting styrene air concentration for the two resins as TD-GC-MS and PID results indicate for the (A) styrene composite and (B) non-styrene composite manufacture

Figure S11. Monitored gas phased styrene response using TD-GC-MS and PID during manufacture of (A) styrene composite (two-layered) and (B) non-styrene composite (two-layered)

S1. Styrene concentration in collected condensates from on-site CIPP installation and multiphase composition of the condensates

S2. Plastic composite manufacturing process and other monitoring device information for temperature and relative humidity

S3. The airflow into the stainless-steel environmental test chamber (ETC)

S4. Controls and decontamination

S5. The detailed analytical methods, quantification, and tube decontamination for thermal desorption samples and statistical methods

S6. Mass conservation formula and numerical approach for the mass conversion model

S7. Headspace testing of emissions from resins and cured composites

S8. Multiple considerations for measuring VOC using the low-cost sensor

S9. Implication and future study

Table S1. List of CIPP associated air contamination incidents. The incidents were found by the authors not included in the 59 incidents reported by Teimouri et al. (2017)[1], 45 incidents reported by Ra et al. (2019)[2], 21 incidents reported by Sendesi et al. (2020)[3], and 14 incidents reported by Noh et al. (2022)[4] in their Supplementary Information file

Incident Location (Year)	Styrene	Description of Events from Reference
Richmond, VA (2022)[5]	nr	"If you went outside in the Fan Tuesday night and smelled something akin to gas, the Richmond Department of Public Utilities (DPU) says there's nothing to worry about. The DPU says sewer rehabilitation work using styrene is being performed in the Fan area. According to the Virginia Department of Health (VDH), styrene is a colorless liquid that evaporates easily and smells sweet, although large quantities can take on a more unpleasant odor. 'The liquid is often used to make styrofoam, disposable utensils, and plastic dishware and is commonly used to reline sewer pipes. If sewers are being relined with polystyrene, the styrene vapors may enter your home until the polymer has completely cured,' the VDH said."
Delray Beach, FL (2022)[6]	nr	PERSONAL COMMUNICATION: Resident contacted Andrew Whelton about suspected chemical exposure at home due to CIPP sewer lining in Delray Beach, FL. Individual reported experiencing chemical exposure symptoms listed on the FLDOH factsheet. When the city was contacted, they indicated there have been multiple CIPP exposure incidents over the years and even 1 incident where people were moved to a hotel.
Grinnell, IA (2022)[7, 8]	nr	MEDIA REPORT & PERSONAL COMMUNICATION: Monday, April 25, 2022 at 11:26 am, the Grinnell Fire Department responded to the 900 block of East for an odor in the basement. Firefighters used an exhaust fan to remove the odor from the basement. Firefighters determined the smell was from work on the city's sewer pipes that are being lined. Assisting at the scene was Midwest Ambulance. Firefighters were on scene for thirty minutes.
Towson, MD (2021)[9]	nr	MEDIA REPORT: An odor in Towson that closed a road Thursday morning (10/14) was determined to have been caused by epoxy. It was nontoxic, according to Baltimore County Emergency Management officials. Initially, firefighters said the odor in the 1000 block of Cowpens Avenue was believed to be natural gas. A hazardous materials crew was called, officials said. "The road is blocked," the Providence Volunteer Fire Company reported before 10:30 a.m. By noon, the fire company reported that investigators determined an epoxy used in a sewer line repair caused the odor in some homes in the area. Nobody was injured, and no schools were affected, according to the Providence Volunteer Fire Company, which used fans to ventilate the impacted residences.
Spooner, WI (2021)[10-12]	nr	MEDIA REPORT & PERSONAL COMMUNICATION: Spooner Middle School will remain closed and remote learning will begin next week after a strong epoxy-like odor prompted an evacuation Wednesday (11/10) and caused 60 staff and students to seek medical treatment. Spooner Area School District Superintendent told WPR a student first reported what they believed to be a gas leak around 8:40 a.m. Wednesday. Staff members investigated the source of the smell, which was near the gym and a classroom wing. Not long after, students and staff began reporting that they weren't feeling well, and the principal ordered an evacuation to a nearby church a half hour later. The district closed the school for the remainder of the day, and students were sent home. The district reported 36 students and 24 staff members have sought medical care since Wednesday, reporting symptoms of nausea and lightheadedness. At

Incident Location (Year)	Styrene	Description of Events from Reference
		least two students were transported to medical facilities outside the area. Officials have yet to identify the cause behind what made people sick. "There's been extensive ongoing carbon monoxide testing, which has all fallen within normal levels," the Superintendent said late Thursday (11/11). "We're working with local health and safety officials and also with a state agency. We will be looking to have more extensive testing conducted to determine the source of that strong epoxy-like odor that made many people sick." Environmental sampling is ongoing, and results of the testing are expected sometime next week.<br>One parent is frustrated she didn’t receive notice from the school about the situation until nearly two hours after the odor was discovered. She said her 12-year-old stepdaughter complained of a headache and nausea when her dad picked her up from school Wednesday. She said "I saw a couple of my friends have to go right to the hospital, get picked up by the EMTs, and headed off in the ambulances. My friend was pretty upset about the whole ordeal, and I am too". "I don't think they handled the situation properly." She said her stepdaughter is doing well, but she wants the district to be more transparent about what’s happening. She and her husband have three kids who attend school within the district. Classes are continuing as scheduled for Spooner Elementary School, Spooner High School and the Washburn County Alternative High School. She worries whether it’s safe for their kids to attend while the investigation continues. She also noted district administration sent out an email Wednesday to parents. According to the email, obtained by WPR, "students were evacuated from the middle school due to an epoxy odor that drifted into the building from the construction on the storm drains." "If it was something to do with the sewers, why are the sewers leaking into the school, for one? And, if it wasn't the sewers, then what was it?"<br>On Friday, the district said the focus of the investigation turned to a product being used as part of work on sewers in the neighborhood. "The sanitary sewer service notification provided to residents prior to the work beginning was not provided to the school. As a result, the school was not able to take the actions that had been suggested to homeowners," according to the Friday press release. No further details were offered on the work being conducted, the product involved, or the actions that were recommended. A woman reached at the district office Friday said officials had no further comment beyond the release.<br>The district is working with local health officials and the Wisconsin Department of Safety and Professional Services as additional environmental testing is taking place. In a statement, the agency's communications director, said it enforces state plumbing codes and regulates employee safety in the public sector. "We have sent inspectors to look into both areas, and we are in the process of reviewing those initial reports. We cannot speculate regarding when we will reach any final conclusions or what those findings will be," he said. The agency will work with those involved if officials identify any concerns that fall under their authority. The district expects virtual learning to resume Monday for the roughly 360 students and 40 staff who work and learn at the middle school in Spooner, which is halfway between Eau Claire and Superior. Remote learning will continue until it's safe for students and staff to

Incident Location (Year)	Styrene	Description of Events from Reference
		return the building. Parents and students will be able to collect any belongings left behind during the evacuation at Spooner Wesleyan Church on Monday.
Montreal, CAN (2021)[13]	nr	MEDIA REPORT: Tenants of a housing cooperative in Montreal were seriously inconvenienced by toxic fumes following rehabilitation work on a sewer line. The odors that normally disappear after a few hours after such work have infiltrated the building and remained there for months. Result: evacuated tenants and a condemned apartment. However, neither the City of Montreal nor the company in charge of the work, wants to take responsibility for the damages they suffer and the expenses that accumulate.
Redfield, SD (2020)[14]	nr	PERSONAL COMMUNICATION: Redfield Fire Department reported that they responded to two separate fire calls on 09/24/2020. The first call was to a residence that had a power cord short-out. The second call was to a residence that could smell odor of Natural Gas. Upon, further investigation on the second call, the city sewer main was in process of getting lined & the epoxy adhesive smell had leached through the sewer.

Notes: nr = not reported in the reference; PERSONAL COMMUNICATION indicates the source contacted co-author Dr. Whelton individually.

Table S2. HAPs which are regulated by the *Clean Air Act* have previously been found in resins or discharged into air.

**List of HAPs Defined by the U.S. EPA that have been Confirmed in CIPP Resins and Emitted into the Environment from CIPP Activity**		
Acetophenone[¥ a]	Ethyl benzene[¥,§ 15-17]	Phthalic anhydride[§ 17]
Aniline	Hydroquinone[§ 3]	Styrene[¥ a,1-3, 15-21]
Benzene[¥ 15]	Maleic anhydride[§ 17]	Styrene oxide[¥,§ a,17]
Chloroform[¥ 15]	Methylene chloride[¥ 2, 15]	Toluene[¥,§ 15, 16]
Cumene[¥,§ a,2, 17]	Methyl ethyl ketone[¥ 15]	Xylene[¥,§ 15, 17]
Dibutyl phthalate (DBP)[¥,§ 1, 17]	Phenol[¥ a, 1, 2]	

Note: [¥]The chemical compounds found in the discharged CIPP air and [§]the compounds found in the uncured resins. The reference from this present study[a], Noh et al. (2022)[16], Teimouri et al. (2017)[1], Ra et al. (2019)[2], Sendesi et al. (2020)[3], Matthews et al. (2020)[15], Li et al. (2019)[17], AirZone (2001)[18], Dusseldorp (2006) [19], NIOSH (2019)[20], Ajdari (2016)[21].

Table S3. Example CIPP use in USA and other countries

Installation Location	Resin Type (monomer)	Resin Mixture Mass (kg)	Pipe Diameter (m)	Pipe Length (m)	Cost (Million USD)
Ontario CAN [22]	Vinyl ester (styrene)	5.4×10^3<br>1.2×10^4<br>6.3×10^4<br>(Total 7.94×10^4)	1.37<br>1.07<br>1.37-1.52	27.4<br>173.7<br>438.9	Not found
Staffordshire ENG [23]	Not found	Each liner weights (7mm thick): 1,5000	1.20	249	0.4*
Mexico City MXN [24]	Isophthalic polyester (styrene)	6.12×10^4	2.13	209.5	Not found
Tilburg NLD [25]	Not found	6.35×10^4	1.98	289	Not found
Culver City, California USA [26]	Vinyl ester (styrene)	3.47×10^5	1.98	1,645.9	60
Napa, California USA [27]	Not found	Not found	Not found	9,817	Not found
Bright Alley, Cincinnati USA [28]	Not found	Not found	0.30	3.05	0.07
Oakley, Cincinnati USA [28]	Not found	Not found	1.52<br>1.83	804.7<br>804.7	12.2
Atlanta, Georgia USA [29]	Not found	Not found	1.2	1,706.9	Not found
Savannah, Georgia USA [30, 31]	Not found	2.72×10^5	2.59	487.7	2.4
Burlington, New Jersey USA [32]	Not found	1.09×10^5	3.05	297.2	0.25
Detroit, Michigan USA [33]	Isophthalic polyester	$> 4.54\times10^5$	2.13 × 2.44 (rectangular)	365.8	50
Malone, New York USA [34]	Not found	Not found	Not found	7,010.4	2.66
Concord, North Carolina USA [35]	Not found	Not found	Not found	6,400.8	Not found
Lake Oswego, Oregon USA [36]	Not found	1.81×10^5	0.41<br>0.91	655.3<br>1,188.7	4.5
Chattanooga, Tennessee USA [37]	Not found	Not found	0.15 ~ 0.45	5,401	Not found
Dallas, Texas USA [38]	Not found	$2.46\times10^4 \sim 2.73\times10^4$	0.6 ~ 2.40	238	Not found
Tyler, Texas USA [39]	Not found	Not found	0.38<br>0.25<br>0.30	1,447.2<br>649.2<br>1,334.1	4.09

Note: Some information was adapted from Noh et al. (2022).[16] The cost is calculated.*

Table S4. Multiple VOCs were emitted into air during and after styrene composite manufacture.

Sample Information	Sampling Time	Chemical Compounds	Chemical Formula	Concentration	% Change in Concentration Compared to the Prior Sampling Time
Middle of Isothermal Curing (10 min after starting isothermal hold)	15 min	Acetophenone [HAP, CI]	C_8H_8O	0.57 ppb	-
		Benzaldehyde [EDR, CI]	C_7H_6O	0.75 ppb	-
		Phenol [EDR, HAP, CI]	C_6H_6O	0.76 ppb	-
		Styrene [CAR, EDR, HAP, CI]	C_8H_8	5.5 ppb	-
End of Isothermal Curing (15 min before halting heating)	15 min	Acetophenone [HAP, CI]	C_8H_8O	0.7 ppb	+22%
		Benzaldehyde [EDR, CI]	C_7H_6O	13.2 ppb	+1,660%
		Styrene [CAR, EDR, HAP, CI]	C_8H_8	19 ppm	+245%
		Styrene oxide [CAR, EDR, HAP, CI]	C_8H_8O	1.65 ppb	-
		1,2,3- TMB [EDR, CI]	C_9H_{12}	2.08 ppb	-
		1,3,5- TMB [EDR, CI]	C_9H_{12}	6.24 ppb	-
During Cooling 1 (10 min after halting heating)	15 min	Acetophenone [HAP, CI]	C_8H_8O	0.57 ppb	-18%
		Benzaldehyde [EDR, CI]	C_7H_6O	2.5 ppb	-81%
		α-methylstyrene [EDR, CI]	C_9H_{10}	0.55 ppb	-
		Styrene [CAR, EDR, HAP, CI]	C_8H_8	7 ppm	-63%
		1,2,3- TMB [EDR, CI]	C_9H_{12}	0.96 ppb	-53%
During Cooling 2 (35 min after halting heating)	10 min	Acetophenone [HAP, CI]	C_8H_8O	5.7 ppb	+900%
		Benzaldehyde [EDR, CI]	C_7H_6O	23.7 ppb	+848%
		Cumene [CAR, EDR, HAP, CI]	C_9H_{12}	35.8 ppb	-
		α-methylstyrene [EDR, CI]	C_9H_{10}	1.7 ppb	+209%
		Phenol [EDR, HAP, CI]	C_6H_6O	1.03 ppb	-
		Styrene [CAR, EDR, HAP, CI]	C_8H_8	51 ppm	+628%
		1,2,3-TMB [EDR, CI]	C_9H_{12}	11.2 ppb	+91%
		1,3,5- TMB [EDR, CI]	C_9H_{12}	29.4 ppb	-

Note: The detection limits are listed in the SI 2.2. The last column (%Change) represents the increase or decrease rate of each compound compared to the previous sampling time. Abbreviations: CAR = carcinogenic compound; EDR = endocrine disruptors; HAP = hazardous air pollutant; CI = chemical irritants; TMB = Trimethylbenzene.

Table S5. Public health and occupational exposure limits of gas phased chemicals

Chemical Name	CAS No.	OEHHA Public Exposure Limit (ppm)[40]	EPA Public Exposure Limit (ppm)[41]	OSHA PEL (ppm)[42]	NIOSH REL (ppm)[43]	ACGIH TLV (ppm)[44]
Acetophenone	98-86-2	N.A.	N.A.	N.A.	N.A.	N.A.
Benzaldehyde	100-52-7	N.A.	N.A.	N.A.	N.A.	N.A.
Cumene	98-82-8	N.A.	50 (10 min; AEGL-1)	(TWA) 50	(TWA) 50	(TWA) 50
α-Methylstyrene	98-83-9	N.A.	N.A.	(TWA) 50 (STEL) 100	(TWA) 50 (STEL) 100	(TWA) 10
Phenol	108-95-2	0.4 (6-hr exposure)	19 (10 min; AEGL-1)	(TWA) 5	(TWA) 5 (Ceiling) 15.6 [15-min]	(TWA) 5
Styrene	100-42-5	4.93 (1-hr exposure)	20 (10 min; AEGL-1)	(TWA) 100	(TWA) 50	(TWA) 20 (STEL) 40
Styrene oxide	96-09-3	N.A.	N.A.	(TWA) 100 (ST) 200	(TWA) 50 (ST) 100	(TWA) 20 (STEL) 40
1,2,3-TMB	526-73-8	N.A.	180 (10 min; AEGL-1)	N.A.	(TWA) 25	(TWA) 25
1,3,5-TMB	108-67-8	N.A.	180 (10 min; AEGL-1)	N.A.	(TWA) 25	(TWA) 25

Note: NIOSH TWA is 10-hr limit; TWA for OSAH and ACGIH based on 8-hr limit. Abbreviations: N.A.=not available, REL=recommended exposure limit, PEL=permissible exposure limits, STEL=short-term exposure limit, TWA=time-weighted average concentration for up to a 10-hour workday during a 40-hour workweek, C=ceiling REL.

Table S6. Chemical volatility and retention time*

Chemical Name	CAS No.	Vapor Pressure (kPa at 25 °C)	Relative Vapor Density (air = 1)	Boiling Point (°C at standard atmospheric pressure, 101 kPa)	Retention Time (min)
Methacrylic acid	79-41-4	0.1	2.97	162.78	4.7
Styrene	100-42-5	0.85	3.6	145	6.76 – 7.0
Toluene	108-88-3	3.8	3.1	110.6	3.8 – 3.9

** retention time under analytical conditions described in section S4*

Table S7. Emitted styrene mass loading during styrene and non-styrene composite manufacture

Sample Name	Overall Manufacture (mg/kg_{resin})		
	Staying (mg/kg)	Isothermal Curing (mg/kg)	Cooling (mg/kg)
Styrene Composite		3,777 ± 132	
	888 ± 111	1,523 ± 405	1,366 ± 242
Non-styrene Composite		57 ± 27	
	14 ± 10	25 ± 12	18 ± 9

Table S8. Statistical summary of the gas-phase styrene concentration comparison measured by PID and TD-GC-MS during styrene and non-styrene composite manufacture

Resin Type for Composite Manufacture	Slope	R^2	Adjusted R^2	MAPE (%)	*p*-value
Styrene based	-58.5	0.66	0.64	251	2.75E-06
Non-styrene based	1,587.1	0.58	0.55	3,406	9.99E-05

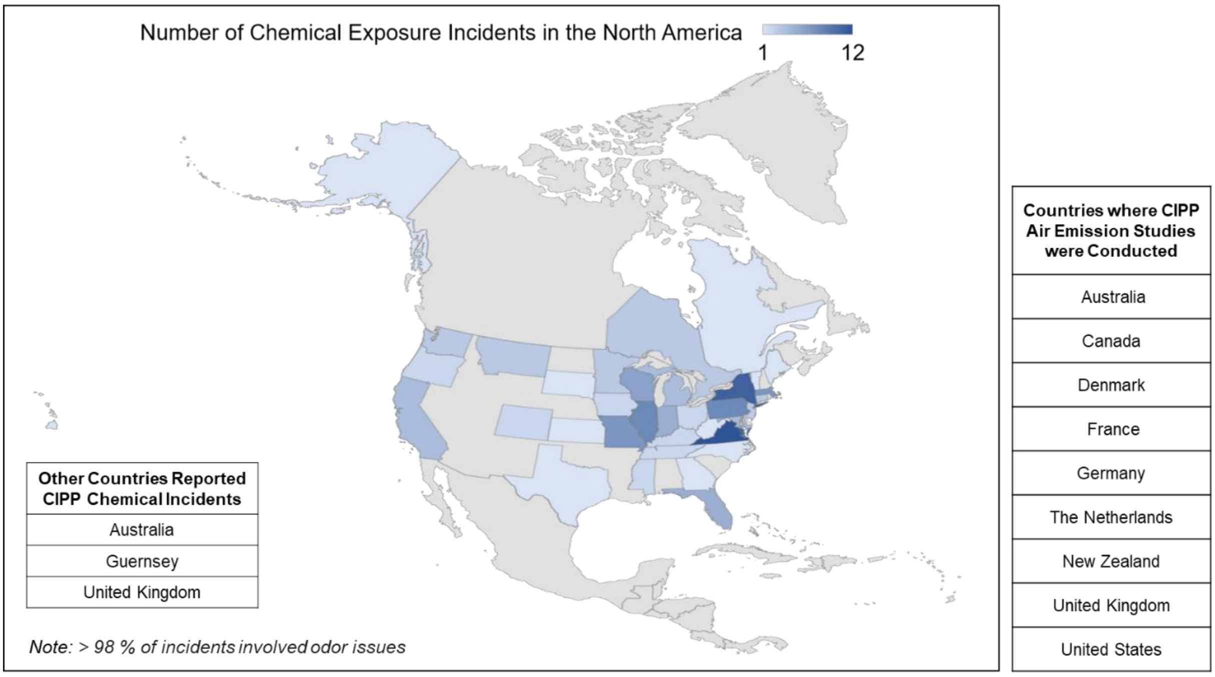

Figure S1. CIPP generated chemical exposure incidents across the world and countries where CIPP air emission studies were conducted. Results shown likely underestimate the number of incidents that were reported publicly. Though a Freedom of Information Act (FOIA) effort in 2017, researchers found that U.S. utilities have had incidents but they are not publicly reported.

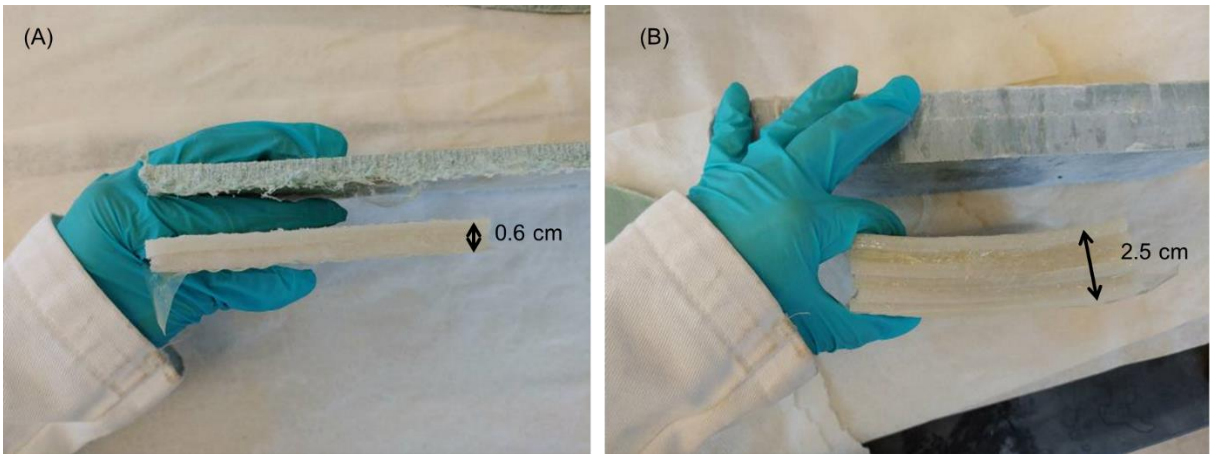

Figure S2. Comparison of lab manufactured composites (bottom; white) and field generated CIPPs (top; blue): [A] thin layer and [B] thick layer.

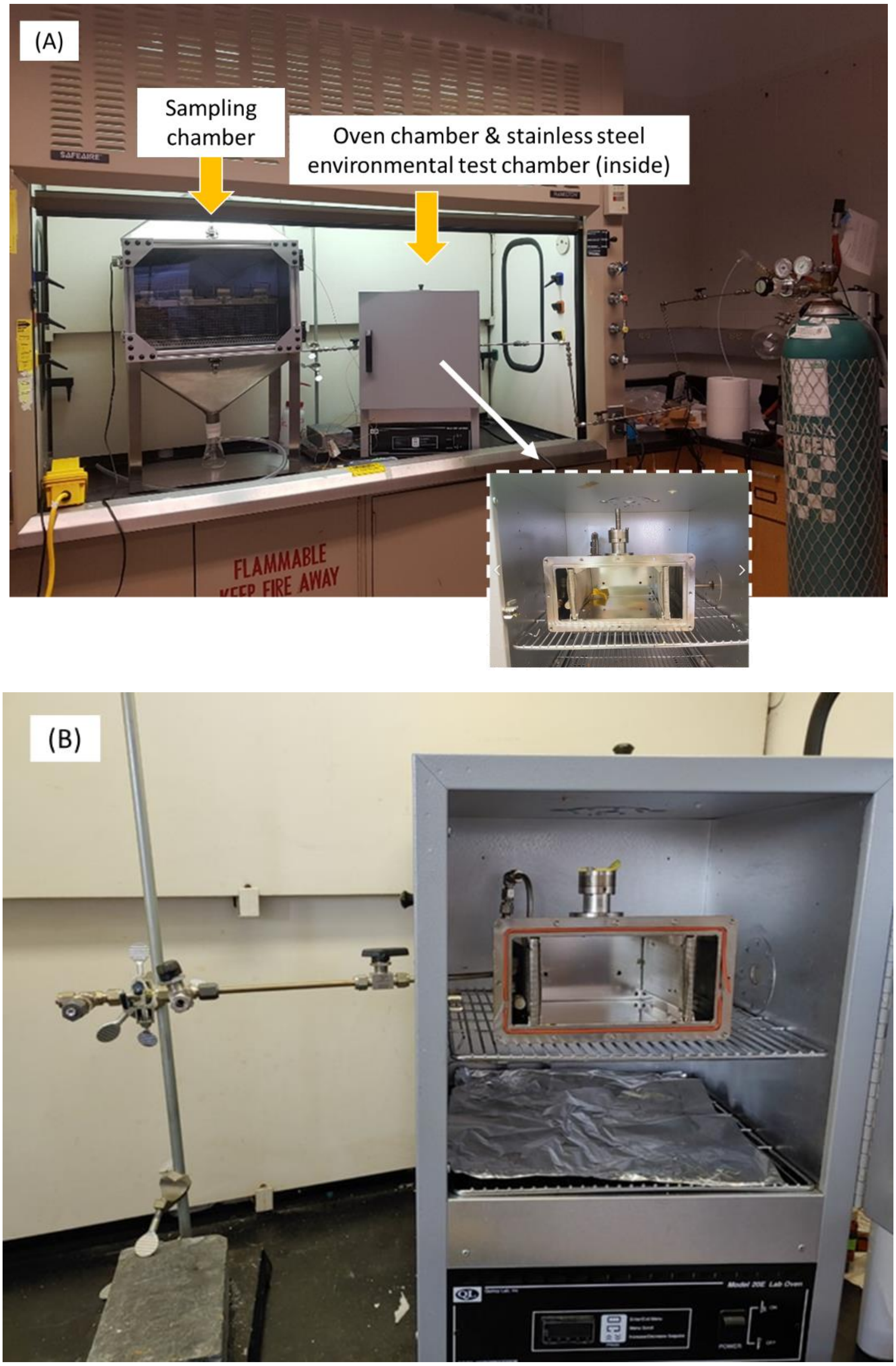

Figure S3. Experimental setup for emitted air collection from [A] sampling chamber and [B] stainless steel environmental test chamber.

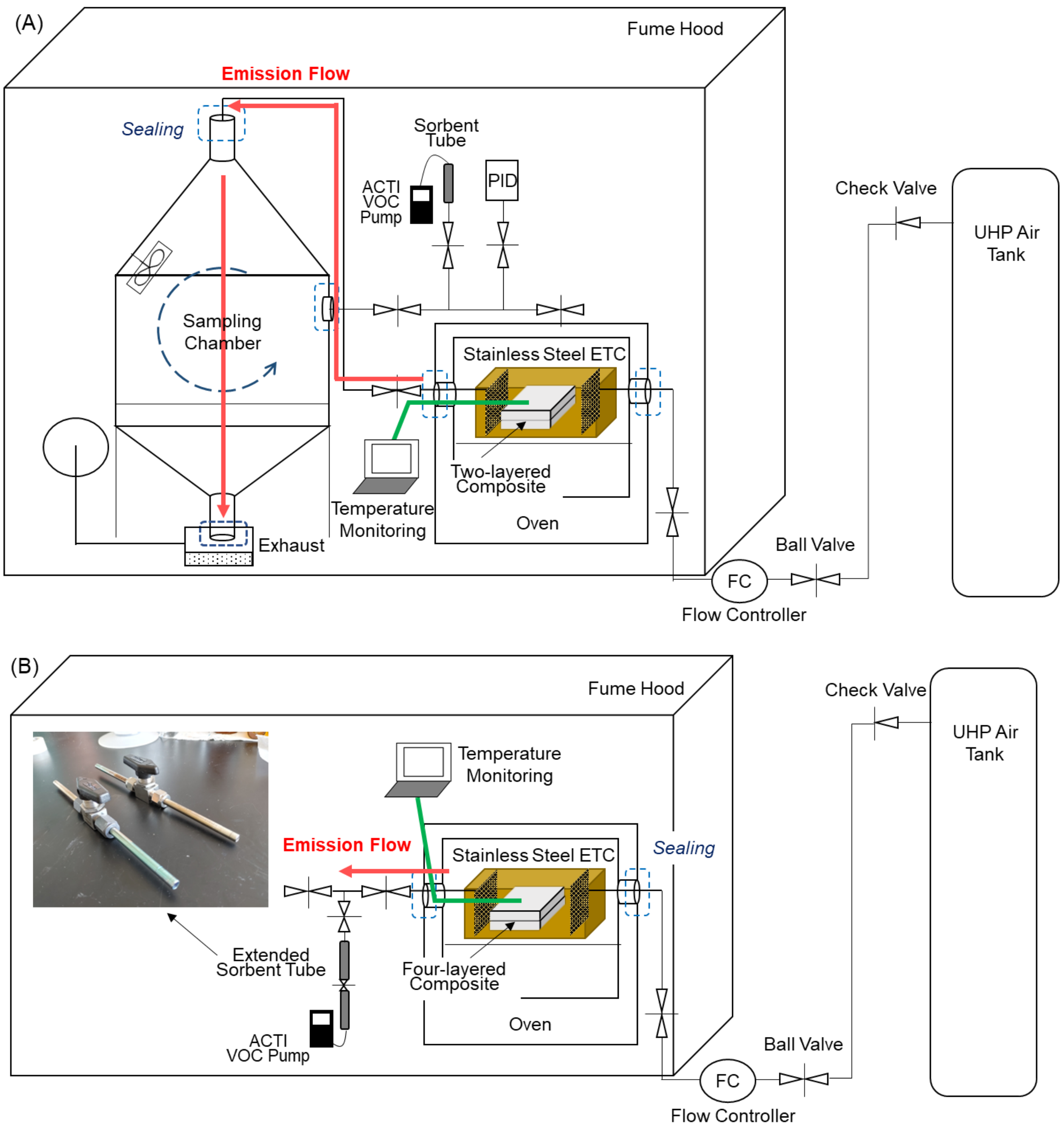

Figure S4. Experimental setup of composite curing and air monitoring for: (A) styrene monitoring in the sampling chamber and (B) multiple chemical monitoring in the ETC. The emission flow is presented as an arrow.

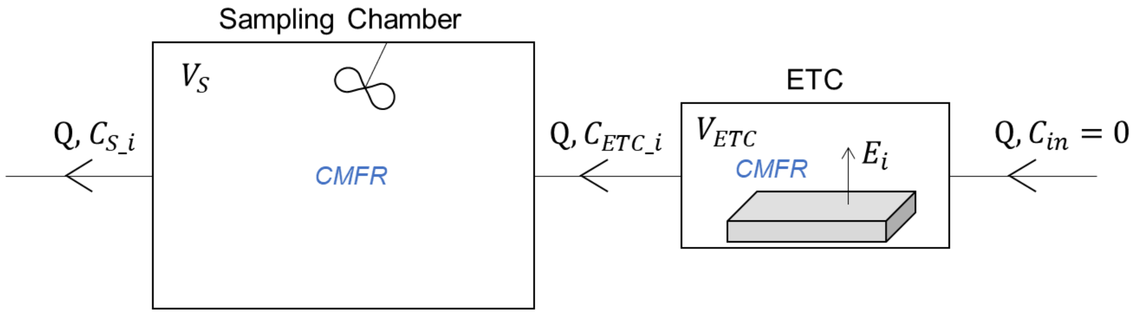

Figure S5. Schematic for the material balance model for gas-phase styrene.

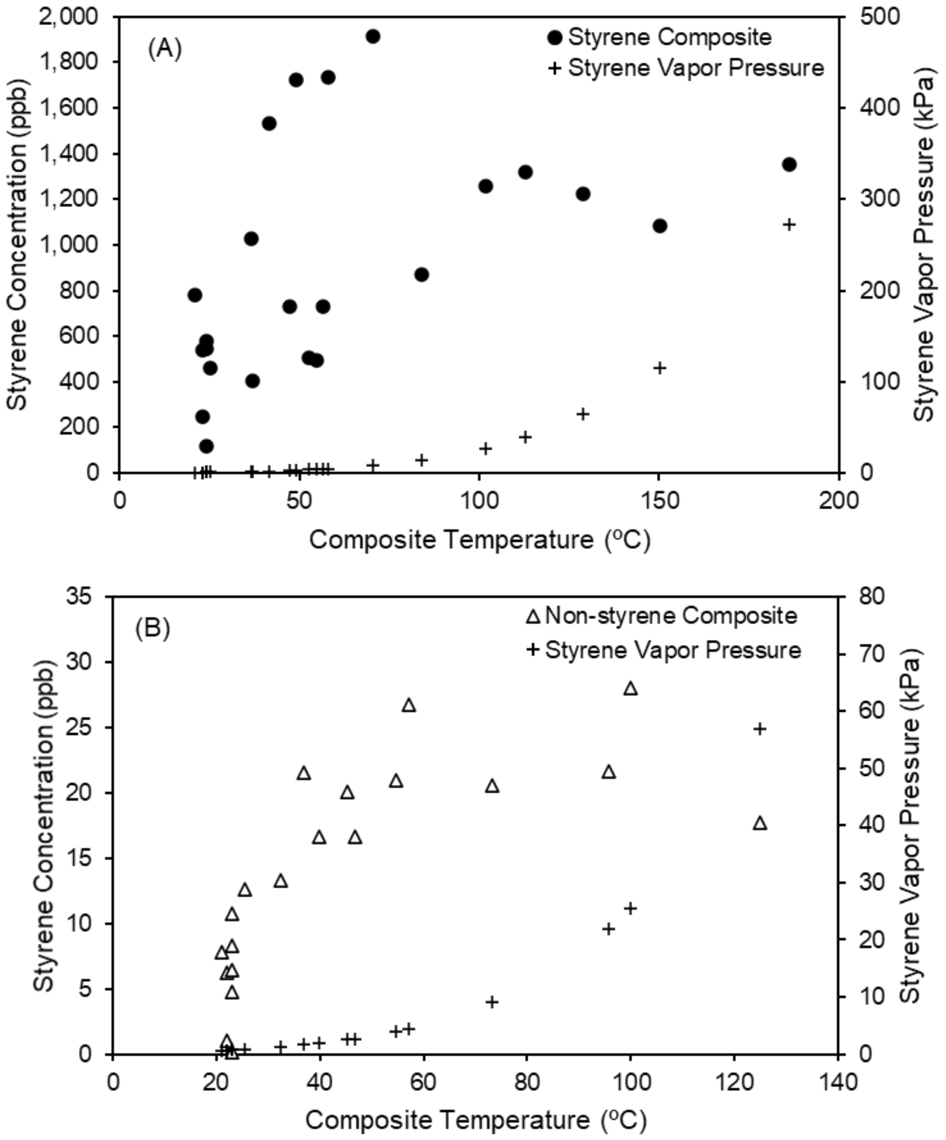

Figure S6. Styrene gas-phase concentrations as measured with TD-GC-MS for (A) styrene composites (two-layered) and (B) non-styrene composites (two-layered) (n=3/assessment) and calculated styrene vapor pressure with respect to composite temperature (°C).

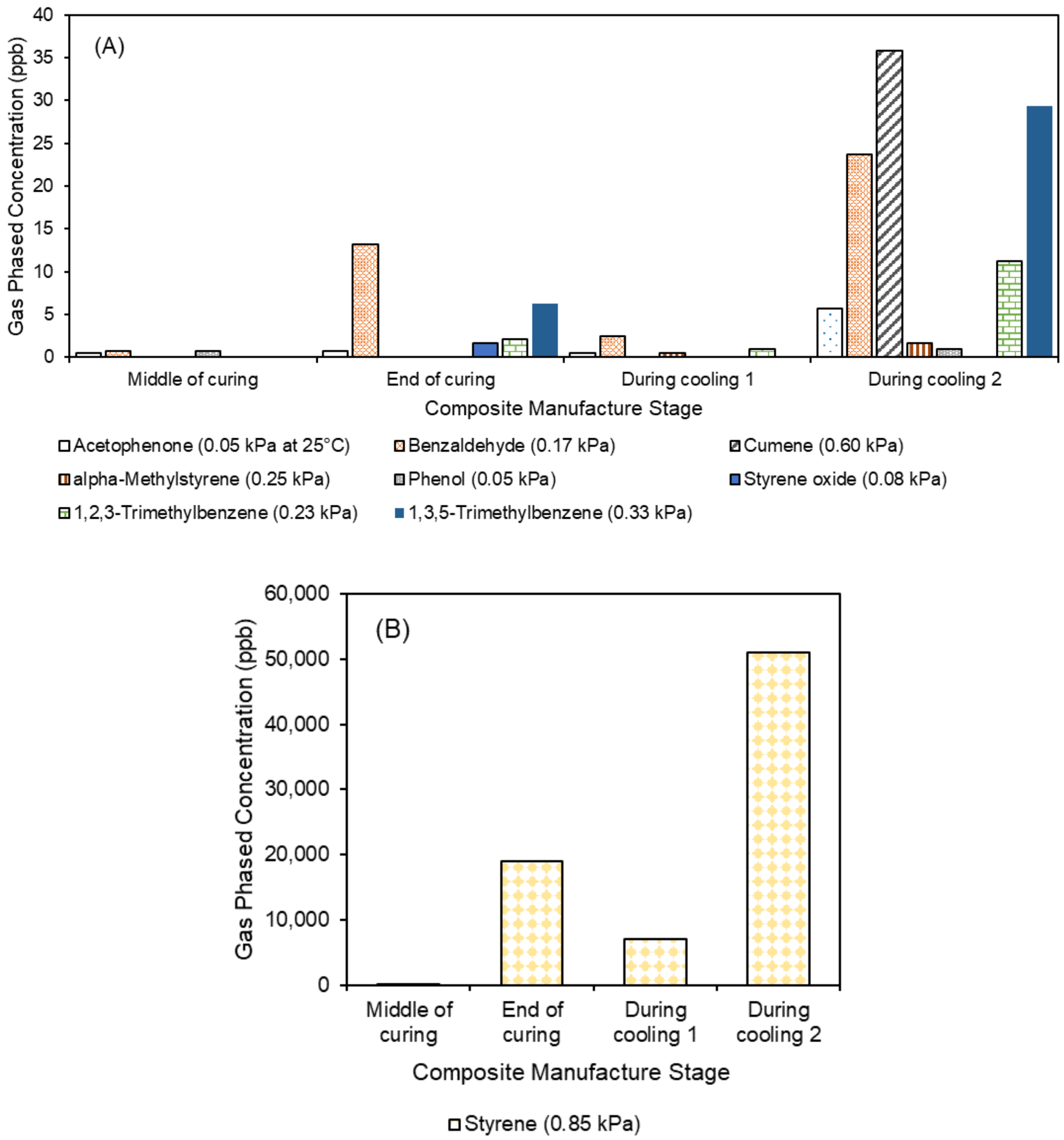

Figure S7. TD-GC-MS measured gas phased chemical concentrations and its volatility (i.e., vapor pressure, kPa at 25 °C) during and after cuing for styrene composite (4-layered).

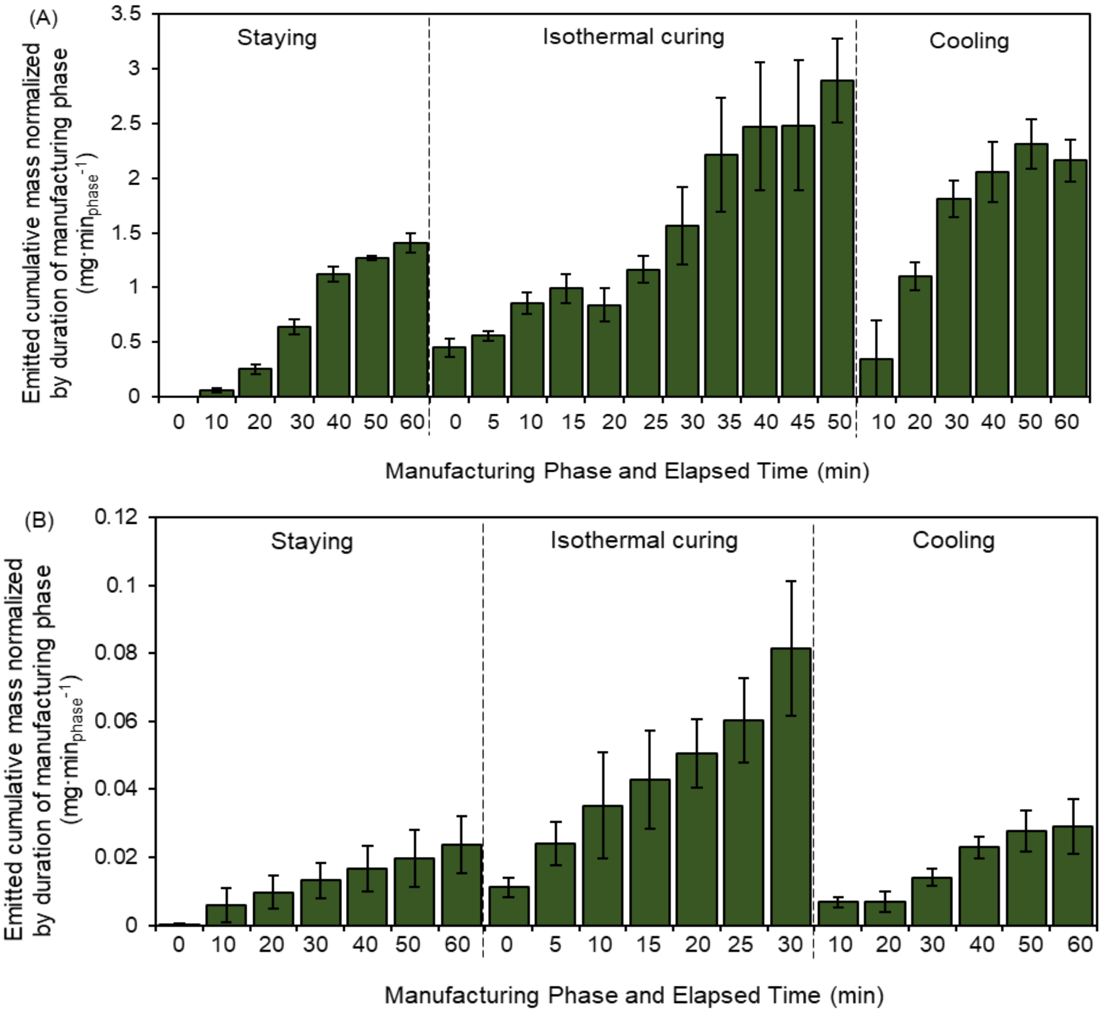

Figure S8. Cumulative mass of gas-phase styrene emission normalized by manufacturing phase duration in the ETC for (A) styrene composites (two-layered) and (B) non-styrene composites (two-layered) manufacture.

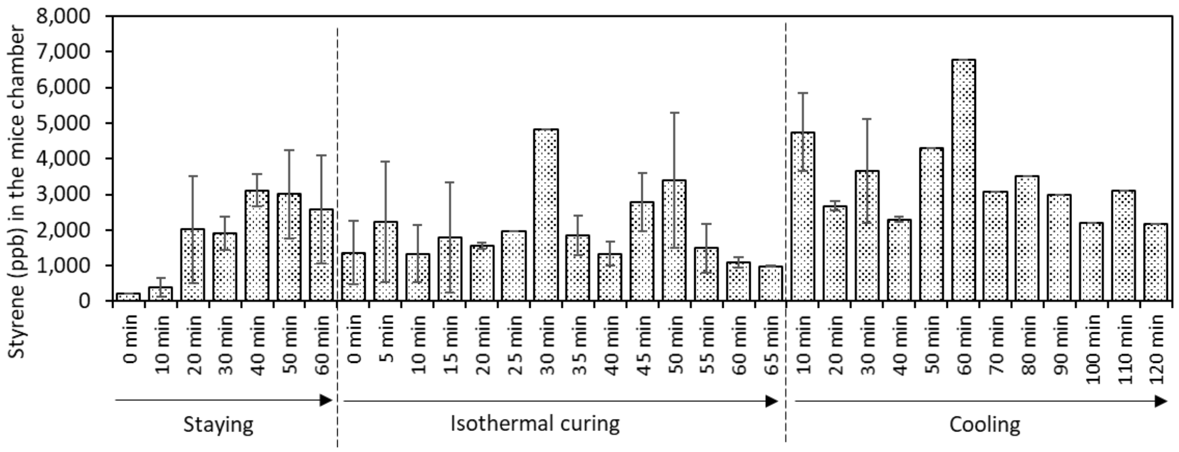

Figure S9. Gas phased styrene monitoring before, during, and after cuing for thick styrene composite (six-layered).

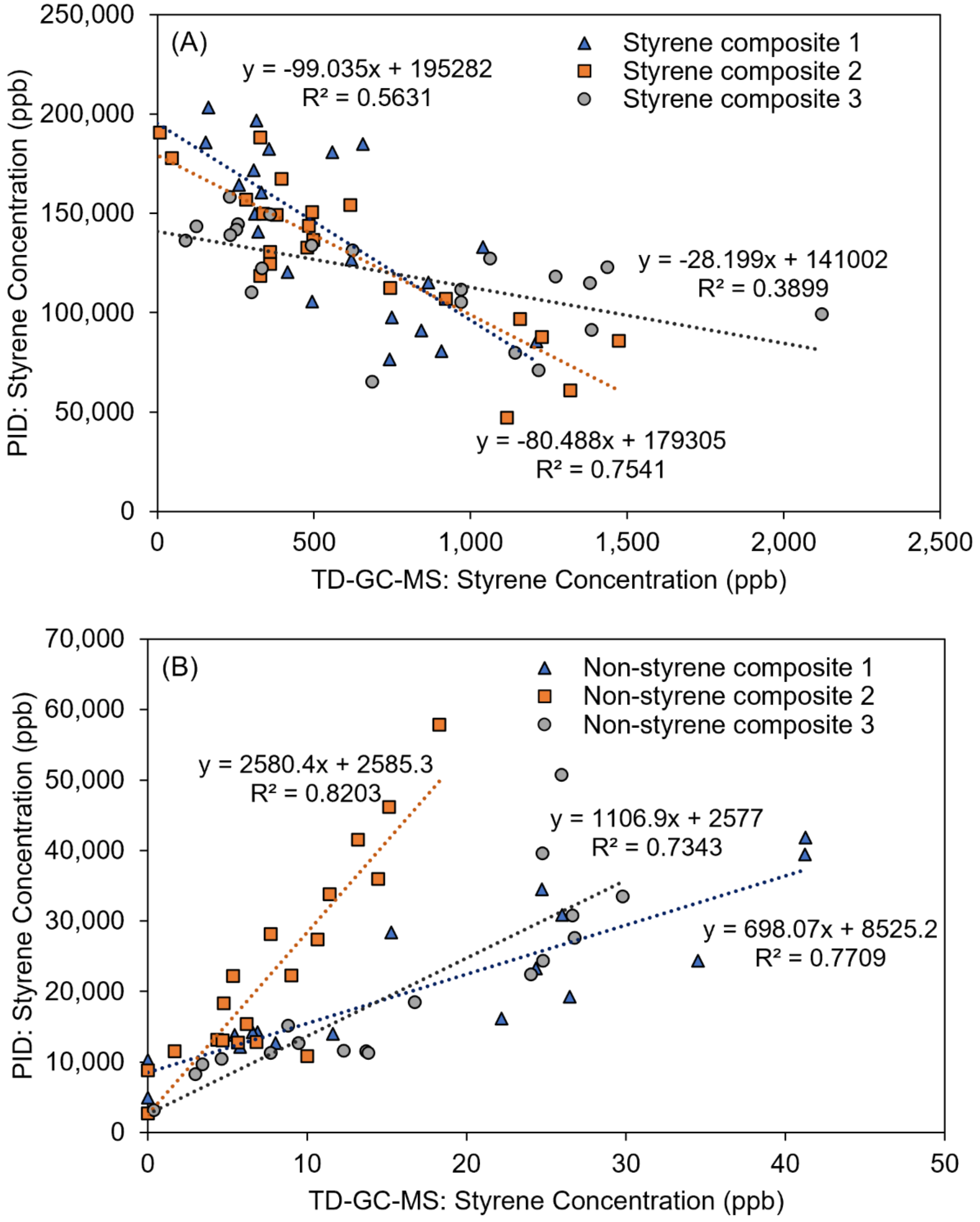

Figure S10. PIDs were not effective in predicting styrene air concentration for the two resins as TD-GC-MS and PID results indicate for the (A) styrene composite and (B) non-styrene composite manufacture. PID signals also greatly exceeded the TD-GC-MS confirmed styrene level.

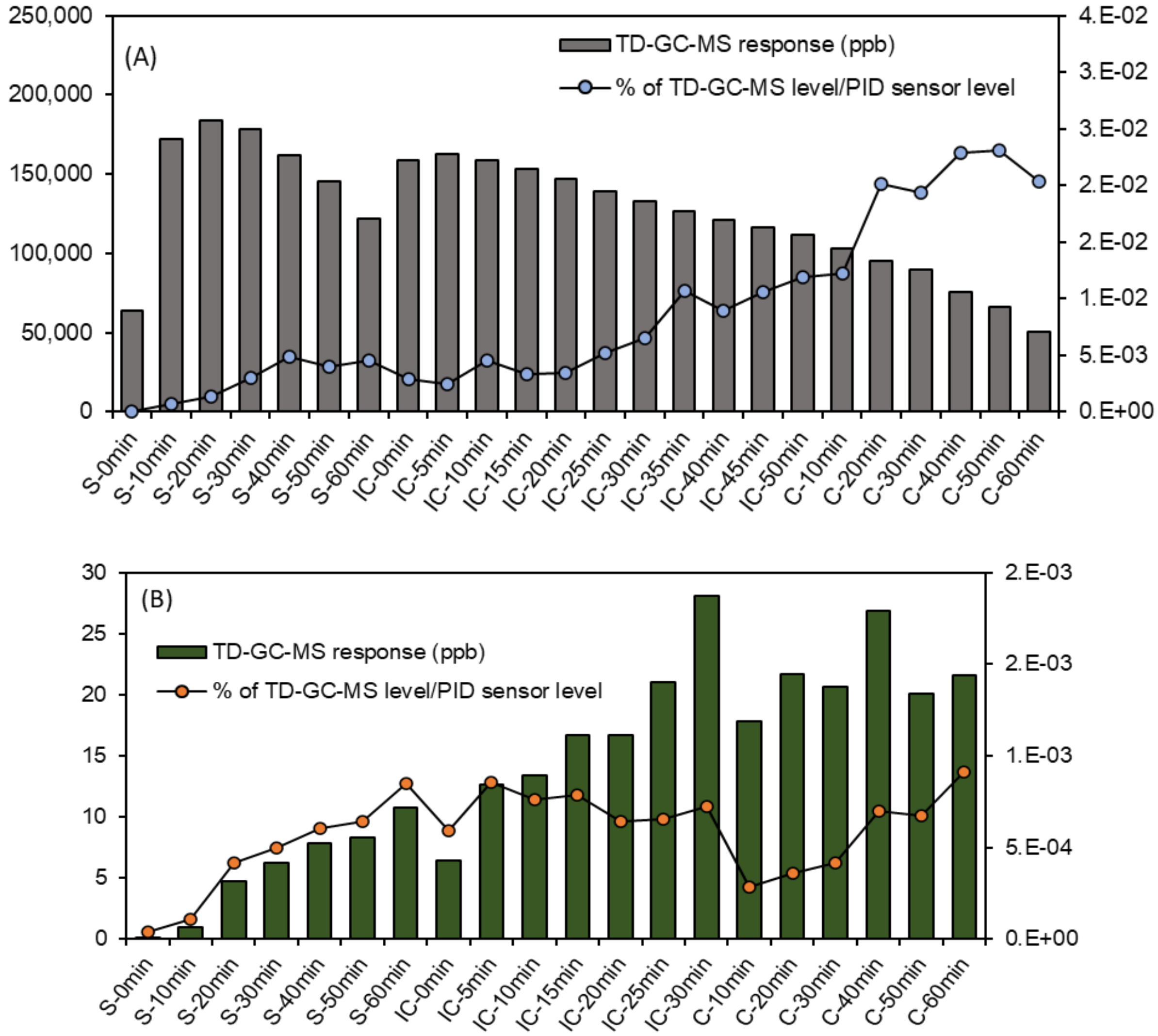

Figure S11. Monitored gas phased styrene response using TD-GC-MS and PID during manufacture of (A) styrene composite (two-layered) and (B) non-styrene composite (two-layered). The abbreviation of 'S', 'IC', and 'C' means 'Staying', 'Isothermal curing', and 'Cooling', separately.

S1. Styrene concentration in collected condensates from on-site CIPP installation and multiphase composition of the condensates.

Total styrene levels found in condensate emitted into the air during styrene based CIPP manufacture monitored by Teimouri et al. (2017).[1] Teimouri et al. (2017) captured air emissions during the manufacture of four styrene-based CIPPs using a emission capture condenser. The captured condensate was then delivered and characterized in the laboratory. It also subjected to a series of dilutions to quantitate the chemical loading. After dilutions and quantitation, the results were used to back calculate the total styrene concentration in the field captured condensate. Styrene levels found in the condensate were 4,329 ppm, 3,590 ppm, 1,819 ppm, and 2,083 ppm. While styrene is only soluble in water at around 300 ppm, the condensate was a multi-phase liquid and some of it was (by volume) organic solvent, particulates (see the image from Teimouri et al. 2017), and nanoplastics (see Morales et al. 2022)[45].

S2. Plastic composite manufacturing process and other monitoring device information for temperature and relative humidity.

Styrene and non-styrene composite plates (10.16 cm × 10.16 cm × 0.6 to 0.8 cm) were manufactured by impregnating a resin mixture into the two-layered pieces of polyester felts. A resin mixture (i.e., resin base + initiators) was prepared following the method recommended by the resin manufacturer.[16] The initiators for styrene are Perkadox® 26 (CAS# 53220-22-7, United Initiators, Inc., OH, USA) and *tert*-butyl peroxybenzoate (TBPB) (CAS# 614-45-9, Sigma-Aldrich, MO, USA). An initiator for non-styrene is cumene hydroperoxide (CAS# 80-15-9, Alfa Aesar, MA, USA).

Temperature profiles of the composite surface in the environmental test chamber (ETC) were measured during the curing process by using a thermocouple thermometer (Item# EW-20250-03, Cole-Parmer®, IL, USA). A handheld thermo-hygrometer (Item# RH85, OMEGA Engineering, CT, USA) was used for measuring the relative humidity (RH).

S3. The airflow into the stainless-steel environmental test chamber (ETC).

Ajdari (2016) measured the volumetric flowrate of steam for the CIPP steam curing.[21] The measurement at the exhaust pipe and/or manhole were challenging because the airflow number on the velocimeter changed drastically.[2, 21] The CIPP industries usually does not report the injected airflow, the Quantor (2006) reported 6,000 m^3/h of air flowrate was estimated in hot water curing.[46]

This study considered CIPP area specific flowrate (m/h) based on the measured flowrate by Ajdari (2016). The pipe length and diameter were 71.6 m and 20.3 cm, separately, and the volumetric flowrate at steam hose during curing stage was measured at 241.26 m^3/h. Based on the calculated CIPP area specific flowrate (5.29 m/h) and the surface area of lab-manufacture composite (two-layered, the total of upper and side of the composite area= 0.014 m^2), the estimated volumetric flowrate for the ETC was 0.08 m^3/h (≈ 1.33 L/min).

S4. Controls and decontamination

As a control test, using UHP air (SG 2001301-1, Indiana Oxygen Co., IN, USA) without any composite, VOC concentrations were monitored to verify background residual contamination before starting each composite emission monitoring. Leakage checks were also conducted before every experiment. The chambers and tubing system were cleaned after each experiment. The chamber sets were cleaned with methylene chloride and acetone at least three times. After that, they were flushed with pressurized air for 10 min and disassembled

components were heated at 200 °C for 48 h except for the chamber itself. After assembling, chamber sets were dried at ambient temperature for 24 h with constant airflow (UHP air, 0.3 L/min). A sorbent tube and PID sampling/measurement were performed to verify the system decontamination.

S5. The detailed analytical methods, quantification, and tube decontamination for thermal desorption samples and statistical methods.

Recently, multi-sorbent beds have been used to increase the absorbent surface area as well as to detect a wide range of VOCs.[47] In this study, it was determined that the emitted styrene concentration was saturated in a single sorbent tube. The extended sorbent tube method was devised with two sorbent tubes connected in series, similar to the multi-bed approach.

For sample quantification, 1 μL of chlorobenzene-d5 (11.57 mg/L) in methylene chloride was injected into the sorbent tubes with collected sample as an internal standard. The prepared sorbent tube samples were processed using an Ultra 2 Series multi-tube autosampler in conjunction with a Unity 2 Series thermal desorption (TD) system (both from Markes International, Inc., CA, USA). The samples were thermally desorbed in the TD, followed by analysis with a GC (2010-Plus, Shimadzu, Inc., MD, USA) and a MS (TQ8040, Shimadzu, Inc., MD, USA). The cold trap temperature in the TD was held at 25ºC and then ramped to 300 ºC at 20 ºC/s, followed by trap hold for 3 min. GC separation was carried out using a HP-5MS capillary column (30 m length, 0.25 mm diameter, 0.25 μm film thickness) with helium as the carrier gas at a flow rate of 1.5 mL/min. The oven temperature program for the GC was as follows: oven temperature of 40 ºC (hold for 2 min) and then ramped to 210 ºC at 15 ºC/min with direct injector mode. The purge flow and column flow were 5 mL/min and 1.5 mL/min, respectively. After analysis by TD-GC-MS, a tube conditioner (TC-20, Markes International,

Inc., CA, USA) was used to decontaminate the sorbent tubes. Decontamination was performed at 325 ºC for 12 h by applying UHP nitrogen gas (99.999%).

The National Institute of Standards and Technology (NIST) database of mass spectra was used for tentatively identifying chemical compounds, and each chemical was quantified using a standard curve. Analytical standards used for TD-GC-MS analysis and their detection threshold are: acetophenone (CAS# 98-86-2, Supelco), 0.48 ppb; benzaldehyde (CAS# 100-52-7, Sigma-Aldrich), 0.55 ppb; cumene (CAS# 98-82-8, Sigma-Aldrich), 0.48 ppb; phenol (CAS# 108-95-2, Sigma-Aldrich), 0.62 ppb; styrene (CAS# 100-42-5, Sigma-Aldrich), 0.56 ppb; styrene oxide 97% (CAS# 96-09-3, Sigma-Aldrich), 0.49 ppb; 1,2,3-trimethylbenzene (CAS# 526-73-8, Sigma-Aldrich), 0.48 ppb; 1,3,5-trimethylbenzene (CAS# 108-67-8, Supelco), 0.48 ppb; a-methylstyrene/ methylethenyl (CAS# 98-83-9, Sigma-Aldrich), 0.50 ppb.

To determine the variance ratio of the regression model, the coefficient of determination (R^2) and the adjusted coefficient of determination (adjusted R^2) were used, and the mean absolute percentage error (MAPE) was also analyzed to evaluate the prediction accuracy.

S6. Mass conservation formula and numerical approach for the mass conversion model.

Since the monitoring chambers were electropolished and background/control measurements were also conducted, the authors assumed that adsorption and desorption for sampling chamber and ETC surfaces were negligible. The mass conversion equations for the ETC and sampling chambers (Equation 1 and Equation 2, separately), EFs (Equation 3), and the cumulative emitted mass per duration of manufacturing phase (CMPD) (Equation 4) in the ETC based on ASTM D6670[48] are as follows:

$$V_{ETC}\frac{dC_{\mathrm{ETC\_i}}}{dt} = \left(C_{in} - C_{ETC\_i}\right) \cdot Q + E_i \qquad \text{(Equation 1)}$$

$$V_S \frac{dC_{S\_i}}{dt} = (C_{ETC\_i} - C_{S\_i}) \cdot Q \qquad \text{(Equation 2)}$$

$$\mathrm{EF} = \frac{\sum_{i=1}^{n} \frac{\mathrm{E_{i+1}+E_i}}{2} \cdot \mathrm{t_i}}{\mathrm{m_{resin}}} \qquad \text{(Equation 3)}$$

$$\mathrm{CMPD} = \frac{\sum_{i=1}^{i} \frac{\mathrm{E_{i+1}+E_i}}{2} \cdot \mathrm{t_i}}{T_{\mathrm{phase}}} \qquad \text{(Equation 4)}$$

where the terms V_{ETC} and V_S (m^3) are the volumes of the ETC and sampling chamber, respectively, C_{in} (mg/m^3) is the influent styrene concentration to the ETC, which is zero, C_{S_i} (mg/m^3) is the styrene concentration inside the sampling chamber at the ith sampling point, C_{ETC_i} (mg/m^3) is the styrene concentration inside the ETC, which was calculated by (Equation 2) using the measured C_{S_i}, Q (m^3/h) is the applied volumetric air flowrate, E_i (mg/h) is the rate of emission (positive) or uptake (negative) at the ith sampling point in the ETC, n (unitless) is the total number of sampling points, t_i (h) is the sampling interval time between i^{th} and $i+1^{th}$ sampling points, EF (mg/kg_{resin}) is the mass of styrene emitted per mass of resin, m_{resin} (kg) is the resin mixture amount for manufacturing the composite, $A_{composite}$ is the composite surface area (m^2), CMPD (mg/h_{phase}) is the cumulative emitted mass per duration of each manufacturing phase, and T_{phase} (h) is the total duration of each manufacturing phase.

S7. Headspace testing of emissions from resins and cured composites.

Sample preparation

For the raw resin samples, four types of samples were analyzed: (1) styrene resin (5 g), (2) styrene resin (4.8 g) with styrene initiators Perkadox® 26 + TBPB (0.2 g), (3) non-styrene resin (5 g), and 4) non-styrene resin (4.9 g) with non-styrene initiator cumene hydroperoxide (0.1 g).

For the cured composite samples, the one-layered styrene and non-styrene composites were manufactured under the same conditions as described in Section 2.1. The cured composites (10.16 cm × 10.16 cm × 0.34 cm) were cut into 8 pieces for one sample. Six types of samples were analyzed: (1) styrene composite (3.13 g of resin mixture), (2) styrene composite soaked in deionized water (5 g), (3) styrene composite after 7 d aging, (4) non-styrene composite (3.13 g of resin mixture), (5) styrene composite soaked in deionized water (5 g), and (6) non-styrene composite after 7 d ventilation. Resin and composite samples numbered above 1, 2, 4 and 5 were placed in the 10 mL headspace vials, sealed and placed in a dark for 24 h at room temperature before headspace analysis with GC-MS (**Figure S12**). The styrene- and non-styrene composite vials undergoing the 7-d ventilation experiment were placed in purified 4 L glass jars. The cap of jar was opened for ventilation every day for 7 days. After that, the sample vials were sealed for 24 h prior to the analysis. All the samples were prepared in triplicate and three blank samples were also prepared.

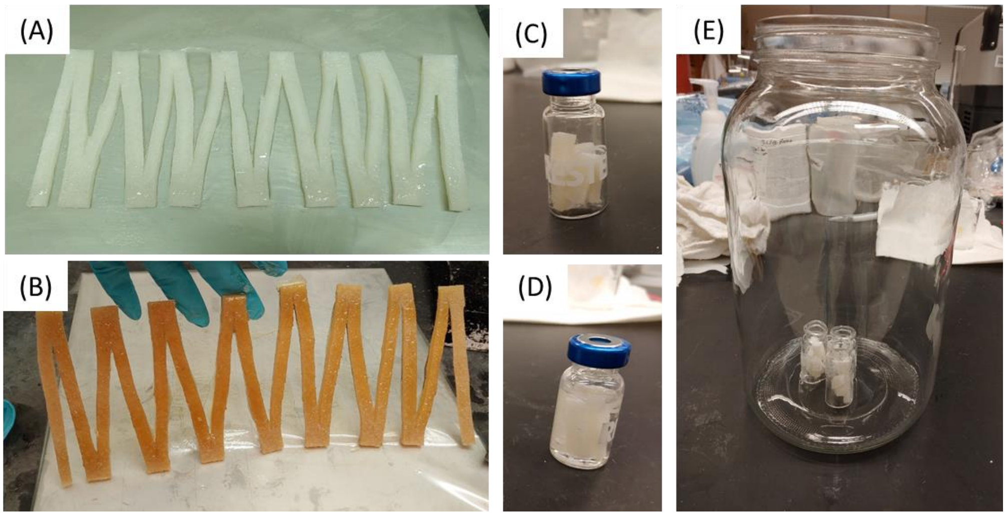

Figure S12. Experimental approach for the headspace analysis: [A] manufactured styrene composite, [B] manufactured nonstyrene composite, [C] styrene composites in the 10 mL headspace vial, [D] styrene composite + deionized water in the 10 mL headspace vial, and [E] styrene composites for 7 d aging in 4 L glass jars.

Analytical approach

All chemicals emitted into the air from the resins and cured composites were analyzed using a GC-MS headspace injection method, which involved vial agitation at 50°C for 10 min. A 500 µL headspace sample was injected using 10:1 split mode via the injection port at 280°C. The oven program was held at 40°C for 4 min before increasing to 121°C at 12°C/min. The quadrupole MS was operated in the scan mode over a m/z range 50-300 amu with the interface temperature set at 250°C, and the ion source held at 220°C. The National Institute of Standards and Technology database of mass spectra was used for tentatively identifying chemical compounds, but only styrene (detection limit: 0.54 mg/kg) and toluene (0.92 mg/kg) was detected.

Pathways by which HAPs enter the environment

Ventilation of the new composite for 7 days resulted in marked reductions in VOC air concentrations. For this method, styrene and toluene were the only VOCs detected in air (**Table S9**). Immersion of the new styrene composite into water for 1 day prompted similar styrene air concentrations above the not immersed composite and the raw resin mixture (i.e., resin + unreacted initiator). Ventilation (7 days) reduced the amount of styrene present in the air above the composite. The non-styrene composite had detectable toluene after manufacture, but toluene was not detected after water exposure or intermittent ventilation. In addition to this, as a future study, site-specific conditions such as the size of newly created CIPPs, airflow, and seasonal effects (i.e., temperature, RH, potential interferent nanoparticle, etc.) should be taken into account to identify emissions and their reductions after CIPP installations.

Table S9. The mass of VOC in air per resin and composite were used to compare the role of post-composite handling on air concentrations

Sample Name	Styrene (mg/kg)	Toluene (mg/kg)
Styrene Resin System and Composite		
Resin, 1d equilibrium (Eq)	178 ± 1.51	-

Resin + Unreacted Initiator, 1d Eq	190 ± 15.5	-
Composite, 1d Eq	227 ± 27.9	-
Composite + Deionized Water, 1d Eq	299 ± 22.2	-
Composite + 7d Intermittent Ventilation, 1d Eq	-	-
Non-styrene Resin System and Composite		
Resin, 1d Eq	0.86 ± 0.11	4.00 ± 0.22
Resin + Unreacted Initiator, 1d Eq	-	5.07 ± 0.31
Composite, 1d Eq	-	2.35 ± 0.44
Composite + Deionized Water, 1d Eq	-	-
Composite + 7d Intermittent Ventilation, 1d Eq	-	-

Note: The detection limits are 0.5 mg/kg of styrene and 0.9 mg/kg of toluene if it is assumed that the material has a same weight as the sample. Abbreviations: mark (–) = Not detected.

S8. Multiple considerations for measuring VOC using the low-cost sensor.

The discovery that styrene levels are not predicted well by the calibrated PID is noteworthy for environmental monitoring as well as public and worker safety protection. To the author’s knowledge, there have been no formal investigations of the factors that influence PID response under CIPP emission conditions. NIOSH previously found high RH (90 %), hot temperature (38 °C), and other conditions (e.g., interferent gas-phase VOCs) influenced the sensitivity and reliability of some PIDs to cyclohexane gas-phase concentration.[49-51] For CIPP projects, PIDs with 10.6 eV lamps have been used.[20] Theoretically, any compound with ionization energy lower than that of the lamp photons (e.g., 10.6 eV) can be measured, which is more than 200 compounds.[52] The manufacturer’s technical data sheet indicates the lamp could be impacted the presence of non-styrene VOCs or a mixture of VOCs.[53, 54] In 2019, NIOSH measured emissions at a few CIPP worksites and reported PID total volatile organic compound (TVOC) concentration, not “styrene”.[20] Spinelle et al. (2017) found that PIDs are not selective to different VOCs.[55] The gas-phase styrene concentrations as measured by the PID and TD-GC-MS in this study provided a low degree of correlation (R^2 = 0.66 and 0.58,

separately) for both styrene and non-styrene composite manufacture (**Table S8**). Another investigator found that the difference between a PID and GC-MS results for gas-phase toluene and VOC mixtures was -32% to 28%.[56] The PID measurement of total concentration of gas-phase hydrocarbon mixtures from construction painting work showed a high correlation with a GC-flame ionization detector (FID) using sorbent tubes containing coconut shell charcoal ($R^2 > 0.94$), even though the PID data underestimated the concentration (3% to 23% less).[57] As the conflicting evidence exists about the reliability of PIDs in ambient and complex atmospheres, their applicability for CIPP process monitoring and investigations should be further conducted (**S8**).

S9. Implication and future study.

Further research on non-styrene CIPP manufacture

The non-styrene composites had detectable chemicals,[16] but emitted fewer detectable VOCs into the air during manufacture. This may have occurred because lower amounts of VOCs were initially present in the resin, but also the analytical methods were not optimal for contaminant identification and quantification. Prior work[16] showed a much lower VOC content in non-styrene resins. Further work is needed to better understand starting materials and pollutants emitted during non-styrene CIPP manufacture, as not all HAPs present in the resins were disclosed on material SDSs.[17]

Further research on various CIPP manufacturing methods and conditions

Composite manufacture in the present study was conducted without steam or hot water, often used for thermal CIPP projects. Because the presence of water may prompt different VOC emission profiles and magnitudes, additional studies are recommended. The emission of VOCs and HAPs in other forms (particulates, dissolved in water vapor droplets) as found in the field[1]

was not considered here, but should be investigated. The role of the resin mass, pipe size, extent of damage, types of inner and outer CIPP coatings, and other factors in the field should be evaluated for quantifying VOC flux. Also, to understand the CIPP VOC flux more accurately, it is necessary to consider the composition of all VOCs emitted from the composite and the thermodynamic behavior of each VOC on the surface and inside the composites. In the present work, the emission factor was calculated based on comparatively brief manufacturer recommended conditions for heating duration (1 h). However, in the field heating for large diameter CIPPs can occur for more than 22 h, which does not include 24 h of cooling.[38] This may prompt significant amounts of VOC and HAP emitted. In some cases, there have been more than 15 CIPPs manufactured in a single city over a few months period so the local and regional air pollution impacts should be estimated.

This study found overtemperature on the composite surface during manufacture. On-site, CIPPs are often installed in a variety of thicknesses (sometimes over 8.6 cm) and material types (corrugated metal, concrete, clay, nodular cast iron, PVC, etc.) depending on the contract and site conditions. All materials have different thermal conductivity, and a large thickness complicates the temperature gradient in the CIPP wall. Noh et al. (2022)[14] investigated the effect of temperature on the mechanical and chemical integrity of styrene and non-styrene CIPP composites on a laboratory scale. However, in-depth temperature gradient studies for various CIPP conditions still need to be performed.

Previous polymer product and water studies indicate that water may catalyze reactions possibly by a factor of several hundred times,[58, 59] plasticize the composite,[60] and even affect the composite's crosslink density and porosity. Water may also accelerate the gas-to-particle conversion of organic gaseous pollutants,[61] and extract chemicals from the resin and composite

and transfer them into the air.[60] No prior studies have investigated this comparative phenomenon between curing types.

Regulators can estimate styrene emission magnitudes for a CIPP project once they know the amount of resin used for thermal CIPP manufacture. Gas-phase emissions can be estimated for periods before, during, and after CIPP manufacture. To estimate the magnitude of other HAPs emitted, including those not listed on safety data sheets, follow-up experiments are recommended. Though, for styrene-based resins the majority of the HAP seems to be styrene, with others as lesser amounts. The magnitude of non-styrene HAP emission (e.g., toluene) may be significant and follow-up work is recommended. Considering the different compound-to-styrene ratios measured in this study and the detectable styrene range at the CIPP worksite (up to 1,800 ppm gas-phase)[15] and 4,300 ppm total styrene (gas and particulate phases),[1] it is possible to estimate the magnitude of each of the VOCs generated by the CIPP installation (**Table S10**).

Table S10. Estimated other compounds levels compared to the styrene

Manufacturing Phase	Confirmed VOC	Measured Conc., (ppb)	Ratio to Styrene	Predicted VOC Levels as ppm when Gas Phased Styrene is …					
				5 ppm	20 ppm	100 ppm	300 ppm	1,000 ppm	1,800 ppm
Middle of Isothermal Curing	Acetophenone	0.57	0.10364	0.52	2.07	10.36	31.09	103.64	186.55
	Benzaldehyde	0.75	0.13636	0.68	2.73	13.64	40.91	136.36	245.45
	Phenol	0.76	0.13818	0.69	2.76	13.82	41.45	138.18	248.73
	Styrene	*5.5*	*1.0*	*5*	*20*	*100*	*300*	*1000*	*1800*
End of Isothermal Curing	Acetophenone	0.7	0.00004	0.00	0.00	0.00	0.01	0.04	0.07
	Benzaldehyde	13.2	0.00069	0.00	0.01	0.07	0.21	0.69	1.25
	Styrene	*19,000*	*1.0*	*5*	*20*	*100*	*300*	*1000*	*1800*
	Styrene oxide	1.65	0.00009	0.00	0.00	0.01	0.03	0.09	0.16
	1,2,3-TMB	2.08	0.00011	0.00	0.00	0.01	0.03	0.11	0.20
	1,3,5-TMB	6.24	0.00033	0.00	0.01	0.03	0.10	0.33	0.59
During Cooling 1	Acetophenone	0.57	0.00008	0.00	0.00	0.01	0.02	0.08	0.15
	Benzaldehyde	2.5	0.00036	0.00	0.01	0.04	0.11	0.36	0.64
	α-MS	0.55	0.00008	0.00	0.00	0.01	0.02	0.08	0.14

	Styrene	*7,000*	*1.0*	*5*	*20*	*100*	*300*	*1000*	*1800*
	1,2,3-TMB	0.96	0.00014	0.00	0.00	0.01	0.04	0.14	0.25
During Cooling 2	Acetophenone	5.7	0.00011	0.00	0.00	0.01	0.03	0.11	0.20
	Benzaldehyde	23.7	0.00046	0.00	0.01	0.05	0.14	0.46	0.84
	Cumene	35.8	0.00070	0.00	0.01	0.07	0.21	0.70	1.26
	α-MS	1.7	0.00003	0.00	0.00	0.00	0.01	0.03	0.06
	Phenol	1.03	0.00002	0.00	0.00	0.00	0.01	0.02	0.04
	Styrene	*51,000*	*1.0*	*5*	*20*	*100*	*300*	*1000*	*1800*
	1,2,3-TMB	11.2	0.00022	0.00	0.00	0.02	0.07	0.22	0.40
	1,3,5-TMB	29.4	0.00058	0.00	0.01	0.06	0.17	0.58	1.04

Notes: This prediction was calculated only based on the compound vs styrene ratio presented in the current study result. Previous literatures reported that the styrene was detected up to 1,825 ppm [15], the styrene concentration range (5 ppm to 1,800 ppm) was chose. *Abbreviations: MS= methylstyrene; TMB = Trimethylbenzene.*

Further research on low-cost sensor for VOC measurement

In the present study, the resins and manufacturer heating conditions (i.e., temperature, time) were applied based on the recommendation of resin manufacturer and composite company. The measured relative humidity (RH) of the exhaust air from the ETC was 7.4 to 26.3 % (**Figure S13**); RH decreased significantly as dry thermal curing progressed and then increased back towards ambient RH range during the cooling phase. RH can decrease as the air can hold more water molecules as the temperature raised due to thermosetting and exothermic reactions. However, it has been reported that the high RH (>75 %) could affect the signal of low-cost VOC sensors, such as photoionization detectors (PID) and electrochemical sensors.[49-51, 62, 63] Because of this, a field condition-based signal adjustment study should be considered for different types of CIPP curing technology and different PIDs. Additional work, such as using proton transfer reaction time-of-flight mass spectrometry (PTR-TOF-MS),[64] is also recommended for real-time monitoring of volatile chemical emissions and exposures at CIPP field sites.

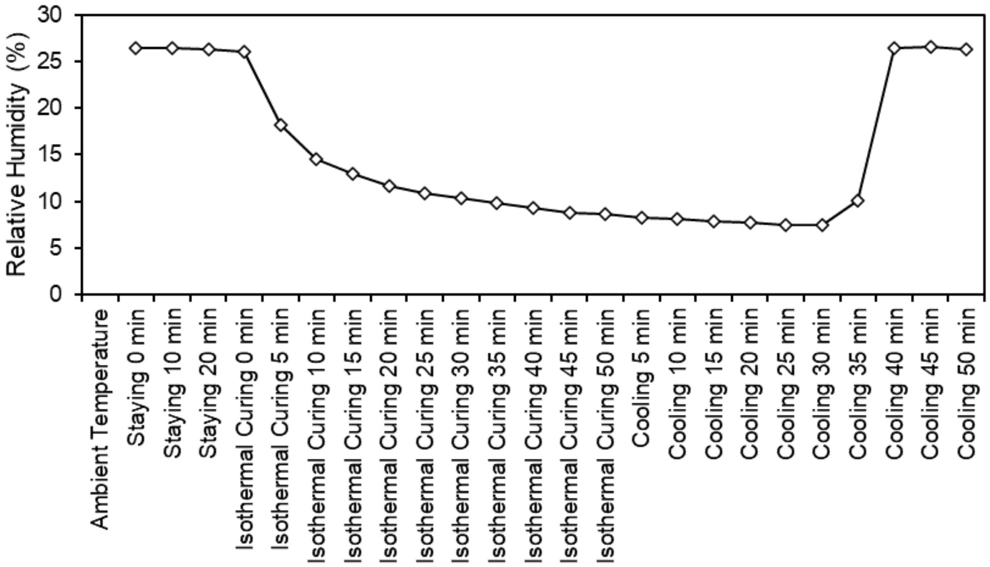

Figure S13. Relative humidity (%) before, during, and after styrene composite manufacture.

Further research on toxicity assessment

Kobos et al. (2019) used styrene-normalized CIPP condensates to determine that trace compounds other than styrene are responsible for differential toxicity.[65] However, exposure toxicity studies of CIPP air waste have not yet been conducted. To investigate CIPP-driven complex volatile chemical wastes, toxicity assessments under controlled conditions should be performed.

References Cited Only in the Supporting Information File.

(1) Teimouri Sendesi, S. M.; Ra, K.; Conkling, E. N.; Boor, B. E.; Nuruddin, M.; Howarter, J. A.; Youngblood, J. P.; Kobos, L. M.; Shannahan, J. H.; Jafvert, C. T., Worksite chemical air emissions and worker exposure during sanitary sewer and stormwater pipe rehabilitation using cured-in-place-pipe (CIPP). *Environ. Sci. Technol. Lett.* **2017**, 4, (8), 325-333. Doi: doi:10.1021/acs.estlett.7b00237.

(2) Ra, K.; Sendesi, S. M. T.; Nuruddin, M.; Zyaykina, N. N.; Conkling, E. N.; Boor, B. E.; Jafvert, C. T.; Howarter, J. A.; Youngblood, J. P.; Whelton, A. J., Considerations for emission monitoring and liner analysis of thermally manufactured sewer cured-in-place-pipes (CIPP). *J. Hazard. Mater.* **2019**, 371, 540-549. Doi: 10.1016/j.jhazmat.2019.02.097.

(3) Sendesi, S. M. T.; Noh, Y.; Nuruddin, M.; Boor, B.; Howarter, J. A.; Youngblood, J. P.; Jafvert, C. T.; Whelton, A., An Emerging Mobile Air Pollution Source: Outdoor Plastic Liner Manufacturing Sites Discharge VOCs into Urban and Rural Areas. *Environ. Sci. Process Impacts* **2020**. Doi: 10.1039/D0EM00190B.

(4) Noh, Y.; Boor, B. E.; Shannahan, J. H.; Troy, C. D.; Jafvert, C. T.; Whelton, A. J., Emergency responder and public health considerations for plastic sewer lining chemical waste exposures in indoor environments. *J. Hazard. Mater.* **2022**, 126832. Doi: 10.1016/j.jhazmat.2021.126832.

(5) K. Hammond, *Richmond, can you smell that? Liquid used in sewer work causes gas-like odor throughout the Fan. 8NEWS*. Available at https://www.wric.com/news/local-news/richmond/richmond-can-you-smell-that-liquid-used-in-sewer-work-causes-gas-like-odor-throughout-the-fan/ **2022**. Richmond, VA. Accessed in January 2023.

(6) Sellers Bird, C. *Personal Communication.* September 23, **2022**. Delray Beach, FL.

(7) Sicard, D., SUBJECT: Records Request: Nov April 2022 air contamination incident and associated investigations, testing, and followup. *Electronic mail from Dan Sicard to Andrew J Whelton*. 2022. Grinnell, IA.

(8) Grinnell Fire Department, Available at https://www.facebook.com/grinnellfd **2022**. Grinnell, IA. Accessed in January 2023.

(9) Janney, E., *Towson Hazmat Call Traced to Substance Used In Repair: Officials*. Available at https://patch.com/maryland/towson/hazmat-call-closes-road-towson-officials October 14, **2021**. Towson, MD. Accessed in January 2023.

(10) Kaeding, D., *Northern Wisconsin school remains closed after odor prompts dozens to seek medical treatment. Wisconsin Public Radio*. Available at https://www.wpr.org/northern-

wisconsin-school-remains-closed-after-odor-prompts-dozens-seek-medical-treatment November 12, **2021**. Spooner, WI. Accessed in January 2023.

(11) DrydenWire.com. *UPDATE: SASD Provides Update On Spooner Middle School Situation.* Available at https://drydenwire.com/news/gas-leak-reported-at-spooner-middle-school/ November 10, **2021**. Spooner, WI. Accessed in January 2023.

(12) Marx, W., SUBJECT: Re: Records Request: Nov 10 air contamination incident at Spooner Middle School and associated investigations, testing, and followup. *Electronic mail from William Marx to Andrew J Whelton*. **2022**. Spooner, WI. Accessed in January 2023.

(13) Tremblay, K., *Styrene Odors. Radio-Canada*. Available at https://ici.radio-canada.ca/tele/la-facture/site/segments/reportage/377409/styrene-toxique-travaux-egout-air-qualite-troubles-voisin November 2, **2021**. Montreal, CAN. Accessed in January 2023.

(14) Redfield Fire Department. *Subject: Re: Redfield, SD epoxy chemical smell incident - September 24, 2020. Electronic mail from Redfield Fire Department to Andrew Whelton*. September 24, **2020**. Redfield, SD.

(15) Matthews, E.; Matthews, J.; Alam, S.; Eklund, S., *NASSCO CIPP Emissions Phase 2: Evaluation of Air Emissions from Polyester Resin CIPP with Steam Cure. Final Report*. For National Association of Sewer Service Companies, Inc. (NASSCO, Inc.) **2020**. Louisiana Tech University, Ruston, LA.

(16) Noh, Y.; Odimayomi, T.; Sendesi, S. M. T.; Younblood, J. P.; Whelton, A. J., Environmental and Human Health Risks of Plastic Composites can be Reduced by Optimizing Manufacturing Conditions. *J. Clean. Prod.* **2022**. Doi: 10.1016/j.jclepro.2022.131803.

(17) Li, X.; Ra, K.; Nuruddin, M.; Sendesi, S. M. T.; Howarter, J. A.; Youngblood, J. P.; Zyaykina, N.; Jafvert, C. T.; Whelton, A. J., Outdoor manufacture of UV-Cured plastic linings for storm water culvert repair: Chemical emissions and residual. *Environ. Pollut.* **2019**, 245, 1031-1040. Doi: 10.1016/j.envpol.2018.10.080.

(18) AirZone, Inc., *A Report on the Monitoring of Styrene in Toronto Homes During the Cured in Place Pipe (CIPP) Process for Sewer Pipe Rehabilitation by Insituform. Project 041-6742* **2001**. Toronto, CAN.

(19) Dusseldorp, A., Schols, E., *Rioolrenovatie met kousmethoden-Achtergronden bij het informatieblad. RIVM rapport 609021038/2006, Bilthoven, RIVM-Rijksinstituut voor Volksgezondheid en Milieu.* **2006**. Amsterdam, NED.

(20) National Institute for Occupational Safety and Health (NIOSH), *Health Hazard Evaluation Report: Evaluation of Exposures to Styrene During Ultraviolet Cured-in-Place-*

Pipe Installation. U.S. Department of Health and Human Services, Centers for Disease Control and Prevention, NIOSH **2019**, *HHE Report No. 2018-0009-3334*, Morgantown, WV.

(21) Ajdari, E. B. Volatile organic compound (VOC) emission during cured-in-place-pipe (CIPP) sewer pipe rehabilitation. **2016**. University of New Orleans, New Orleans, LA.

(22) Underground Construction. Automotive Factory's Pipes Repaired with CIPP Technology. *Underground Construction*. Available at https://ucononline.com/magazine/2014/january-2014-vol-69-no-1/rehabilitation/automotive-factory-s-pipes-repaired-with-cipp-technology **2014**. Benicia, CA. Accessed in January 2023.

(23) Lanes Group. Lanes installs 'biggest ever' liners twice in one week. *Lanes Group*. Available at https://www.lanesfordrains.co.uk/company-news/news/lanes-installs-biggest-ever-liners-twice-in-one-week/ **2021**. Leeds, GBR. Accessed in January 2023.

(24) Trenchless Technology. Sewer Rehab in Mexico City. *Trenchless Technology*. Available at https://trenchlesstechnology.com/sewer-rehab-in-mexico-city/ **2018**. Bernardsville, NJ. Accessed in January 2023.

(25) Uppelschoten, Edwin. Super-sized sewer project demands CIPP method. *WaterWorld*. Available at https://www.waterworld.com/home/article/16200384/supersized-sewer-project-demands-cipp-method **2003**. Tulsa, OK. Accessed in January 2023.

(26) Steinbacher, A. Huge CIPP Project in California. *Trenchless Technology*. Available at https://trenchlesstechnology.com/huge-cipp-project-in-california/ **2009**. Bernardsville, NJ. Accessed in January 2023.

(27) Gregory, R., *Napa Sanitation District*. Available at https://www.facebook.com/ryan.gregory.33886/posts/pfbid0HxSQaggTeu4eg8cAtugr3vkw2BpU2nG8Zf1LuQuLCnyQu41ZUsr4aA2ayZRKAF3bl **2018**. Napa, CA. Accessed in January 2023.

(28) Midwest Mole Company. Cincinnati Embraces Trenchless to Maintain, Update Aging Infrastructure. *Midwest Mole Company*. Available at https://midwestmole.com/cincinnati-embraces-trenchless-to-maintain-update-aging-infrastructure/ **2015**. Greenfield, IN. Accessed in January 2023.

(29) Smith, K., *Sewer line work to start near Emory's Atlanta campus*. Available at https://news.emory.edu/stories/2022/01/er_dekalb_lullwater_sewer_town_hall_11-01-2022/story.html **2022**. Atlanta, GA. Accessed in January 2023.

(30) Gilbert, A. Savannah Bolton Street stormwater drainage project complete. *Savannah Now.* Available at https://www.savannahnow.com/news/20190703/savannah-bolton-street-stormwater-drainage-project-complete **2019**. Savannah, GA. Accessed in January 2023.

(31) Curl, E. Savannah to employ modern tech to shore up 19th century brick stormwater line. *Savannah Now.* Available at https://www.savannahnow.com/news/20180805/savannah-to-employ-modern-tech-to-shore-up-19th-century-brick-stormwater-line **2018**. Savannah, GA. Accessed in January 2023.

(32) Underground Construction. Record Breaking 120-Inch CIPP. *Underground Construction*. Available at https://ucononline.com/magazine/2016/november-2016-no-71-vol-11/features/record-breaking-120-inch-cipp **2016**. Benicia, CA. Accessed in January 2023.

(33) Dayton, S. Born from Opportunity: Florida contractor draws on multiple technologies to tackle a wide range of lining challenges. *Plumber™*. Available at https://www.plumbermag.com/how-to-articles/pipe_cured_sewer_relining/born_from_opportunity **2012**. Accessed in January 2023.

(34) Dumas, A., *Village of Malone - Sanitary Sewer Rehabilitation Project*. Available at https://www.facebook.com/andrea.dumas.9/posts/pfbid02jXjGN6bHLiHFqsLnBiujVCWGKCDmg6JCnHfXZDfFJyqF2NAETm5a3HoRv22ShrWsl **2019**. Malone, NY. Accessed in January 2023.

(35) Whisenant, D., *Annual rehabilitation work on City of Concord sewer lines to begin Monday, June 13*. Available at https://www.wbtv.com/2022/06/08/annual-rehabilitation-work-city-concord-sewer-lines-begin-monday-june-13/ **2022**. Concord, NC. Accessed in January 2023.

(36) Farr, A. CIPP Project. *Trenchless Technology*. Available at https://trenchlesstechnology.com/cipp-project/ **2010**. Bernardsville, NJ. Accessed in January 2023.

(37) The Chattanoogan.com, *Hamilton County WWTA Currently Rehabilitating East Ridge Sewage Lin*es. Available at https://www.chattanoogan.com/2018/11/8/379588/Hamilton-County-WWTA-Currently.aspx **2018**. Chattanooga, TN. Accessed in January 2023.

(38) Matthews, J.C., Large-diameter sewer rehabilitation using a fiber-reinforced cured-in-place pipe. *Pract. Period. Struct. Des. Cons*. **2015**, 20, (2), 04014031. Doi: 10.1061/(ASCE)SC.1943-5576.0000231.

(39) KLTV Digital Media Staff, *Tyler City Council approves upgrades for sewer lines in north end of the city*. Available at https://www.msn.com/en-us/entertainment/news/tyler-

city-council-approves-upgrades-for-sewer-lines-in-north-end-of-the-city/ar-AA10wfBs **2022**. Tyler, TX. Accessed in January 2023.

(40) Office of Environmental Health Hazard Assessment (OEHHA). *Air Toxics Hot Spots Program Risk Assessment Guidelines Part III: Technical Support Document for the Determination of Noncancer Chronic Reference Exposure Levels to solicit public comment*. https://oehha.ca.gov/air/crnr/air-toxics-hot-spots-program-risk-assessment-guidelines-part-iii-1999 **1999**. Sacramento, CA. Accessed in January 2023.

(41) U.S. Environmental Protection Agency (USEPA). *Access Acute Exposure Guideline Levels (AEGLs)*. Available at https://www.epa.gov/aegl/access-acute-exposure-guideline-levels-aegls-values#:~:text=AEGL%20Values%20%20%20%20CAS%20NO%20,%20Methyl%20hydrazine%20%20183%20more%20rows%20 **2022**. Washington, DC. Accessed in January 2023.

(42) Occupational Safety and Health Administration (OSHA). *Permissible Exposure Limits-OSHA Annotated Table Z-2*. Available at https://www.osha.gov/dsg/annotated-pels/tablez-2.html **2022**. Washington, DC. Accessed in January 2023.

(43) National Institute for Occupational Safety and Health (NIOSH). *NIOSH Pocket Guide to Chemical Hazards*. Available at https://www.cdc.gov/niosh/npg/npgsyn-a.html **2022**. Washington, DC. Accessed in January 2023.

(44) American Conference of Governmental Industrial Hygienists (ACGIH). *ACGIH. TLV/BEI Guidelines.* Available at https://www.acgih.org/science/tlv-bei-guidelines/2022. **2022**. Cincinnati, OH. Accessed in January 2023.

(45) Morales, A. C., Tomlin, J.M., West, C.P., Rivera-Adorno, F.A., Peterson, B.N., Sharpe, S.A., Noh, Y., Sendesi, S.M., Boor, B.E., Howarter, J.A. and Moffet, R.C., Atmospheric emission of nanoplastics from sewer pipes repair. *Nat. Nanotechnol*. **2022**. Doi: 10.1038/s41565-022-01219-9.

(46) Quantor, *Development of styrene vapour during the renovation of sewer pipes, Report AK-06-006. RIVM (Rijksinstituut voor Volksgezondheid en Milie; Netherlands National Institute for Public Health and the Environment)* **2006**. Amsterdam, NED.

(47) Gallego, E., Roca, F. J., Perales, J. F., Guardino, X. Comparative study of the adsorption performance of a multi-sorbent bed (Carbotrap, Carbopack X, Carboxen 569) and a Tenax TA adsorbent tube for the analysis of volatile organic compounds (VOCs). *Talanta*. **2010**, 81(3), 916-924. Doi: 10.1016/j.talanta.2010.01.037.

(48) American Society for Testing and Materials international (ASTM International). *ASTM D6670-18 Standard Practice for Full-Scale Chamber Determination of Volatile Organic Emissions from Indoor Materials/Products*. **2007**. West Conshohocken, PA.

(49) Coffey, C.; LeBouf, R.; Lee, L.; Slaven, J.; Martin, S., Effect of calibration and environmental condition on the performance of direct-reading organic vapor monitors. *J. Occup. Environ. Hyg.* **2012**, 9 (11), 670-680. Doi: 10.1080/15459624.2012.725015.

(50) LeBouf, R. F.; Slaven, J. E.; Coffey, C. C., Effect of calibration environment on the performance of direct-reading organic vapor monitors. *J. Air Waste Manag. Assoc.* **2013**, 63 (5), 528-533. Doi: 10.1080/10962247.2013.772926.

(51) LeBouf, R. F.; Coffey, C. C., Effect of interferents on the performance of direct-reading organic vapor monitors. *J. Air Waste Manag. Assoc.* **2015**, 65 (3), 261-269. Doi: 10.1080/10962247.2014.986308.

(52) Honeywell International, Inc., Handheld PID Monitors- User's Guide. Available at https://www.lesman.com/amfile/file/download/file/3765/product/3819/ **2018**. Charlotte, NC. Accessed in January 2023.

(53) RAE Systems, Inc., Correction Factors, Ionization Energies*, And Calibration Characteristics. Available at https://gastech.com/sites/default/files/RAE%20Systems%20Technical%20Note%20106%20v14%20Correction%20Factors.pdf **2010**. Sunnyvale, CA. Accessed in January 2023.

(54) Henderson, R. E., *Questions, Myths and Misconceptions: About using photoionization detectors. Health & Safety*. Access in **2022**.

(55) Spinelle, L., Gerboles, M., Kok, G., Persijn, S., Sauerwald, T., Review of portable and low-cost sensors for the ambient air monitoring of benzene and other volatile organic compounds. *Sensors*. **2017**, 17 (7), 1520. Doi: 10.3390/s17071520.

(56) ISO. ISO 16000-29:2014 Indoor air — Part 29: Test methods for VOC detectors. Available at https://www.iso.org/standard/55227.html2014. **2014.** Geneva, CHE. Accessed in January 2023.

(57) Coy, J. D.; Bigelow, P. L.; Buchan, R. M.; Tessari, J. D.; Parnell, J. O., Field evaluation of a portable photoionization detector for assessing exposure to solvent mixtures. *Am. Ind. Hyg. Assoc. J.* **2000**, 61 (2), 268-274. Doi: 10.1080/15298660008984536.

(58) Butler, R. N., Coyne, A. G., Organic synthesis reactions on-water at the organic–liquid water interface. *Org. Biomol. Chem.* **2016**, 14, (42), 9945-9960. Doi: 10.1039/C6OB01724J.

(59) Guo, D., Zhu, D., Zhou, X., Zheng, B., Accelerating the “On Water” Reaction: By Organic–Water Interface or By Hydrodynamic Effects? *Langmuir*. **2015**, 31, (51), 13759-13763. Doi: 10.1021/acs.langmuir.5b04031.

(60) Akiya, N., Savage, P. E., Roles of water for chemical reactions in high-temperature water. *Chem. Rev.* **2002**, 102, (8), 2725-2750. Doi: 10.1021/cr000668w.

(61) Wu, Z., Chen, J., Wang, Y., Zhu, Y., Liu, Y., Yao, B., Zhang, Y., Hu, M., Interactions between water vapor and atmospheric aerosols have key roles in air quality and climate change. *Natl. Sci. Rev*. **2018**, 5, (4), 452-454. Doi: 10.1093/nsr/nwy063.

(62) Soo J-C., Lee E. G., LeBouf R. F., Kashon M. L., Chisholm W, Harper M. Evaluation of a portable gas chromatograph with photoionization detector under variations of VOC concentration, temperature, and relative humidity. *J. Occup. Environ. Hyg*. **2018**, 15(4), 351-360. Doi: 10.1080/15459624.2018.1426860.

(63) Gebicki J. Application of electrochemical sensors and sensor matrixes for measurement of odorous chemical compounds. *Trends Analyt Chem*. **2016**, 77, 1-13. Doi: 10.1016/j.trac.2015.10.005.

(64) Jiang, J., Ding, X., Isaacson, K.P., Tasoglou, A., Huber, H., Shah, A.D., Jung, N. and Boor, B.E., Ethanol-based disinfectant sprays drive rapid changes in the chemical composition of indoor air in residential buildings. *J. Hazard. Mater. Lett.* **2021**, 2, 100042. Doi: 10.1016/j.hazl.2021.100042.

(65) Kobos, L., Teimouri Sendesi, S. M., Whelton, A. J., Boor, B. E., Howarter, J. A., Shannahan, J., In vitro toxicity assessment of emitted materials collected during the manufacture of water pipe plastic linings. *Inhal. Toxicol*. **2019**, 31, (4), 131-146. Doi: 10.1080/08958378.2019.1621966.
